# One Health preparedness and response for mosquito-borne viruses: a stakeholder- and social network-analysis in the Netherlands

**DOI:** 10.1186/s12889-025-21539-4

**Published:** 2025-01-24

**Authors:** Pauline A. de Best, H. Broekhuizen, R. S. Sikkema, M. P. G. Koopmans, A. Timen

**Affiliations:** 1 Viroscience, Erasmus University Medical Center, Rotterdam, 3015 GD The Netherlands; 2 National Institute for Public Health and the Environment (RIVM), Bilthoven, 3721 MA The Netherlands; 3 Department of Social Sciences, Health and Society, Wageningen University & Research, Wageningen, 6706 KN The Netherlands; 4 Department of Primary and Community Care, Radboud University Medical Center, Nijmegen, 6525 GA The Netherlands; 5 Athena Institute, VU University Amsterdam, Amsterdam, 1081 HV The Netherlands

**Keywords:** Preparedness and response, Vector-borne diseases, One health, Inter-sectoral collaboration, Stakeholder network, Social network analysis

## Abstract

**Background:**

The emergence of mosquito-borne viruses (MBVs) in Europe emphasizes the need for preparedness and response plans. This requires knowledge integration and collaboration across the human, animal, vector, and environmental health domains, aligning with the One Health approach. Despite the importance of a One Health approach, engaging stakeholders from each domain remains challenging. This study aims to identify stakeholders in the field of preparedness and response to MBVs in the Netherlands and map collaborations, knowledge- and information-sharing between these stakeholders, their domains and governance levels. In addition, we aim to identify bottlenecks in these networks and uncover underlying reasons.

**Methods:**

This study combined stakeholder- and social network analysis. Stakeholders were identified through document analysis and snowballing. Semi-structured interviews were conducted with eligible stakeholders. Stakeholders’ collaborations, dependencies, and their roles in MBV preparedness and response were discussed. Interviewees not currently active in MBV policy were given the opportunity to share their experiences regarding ‘zoonotic infectious diseases’ or ‘healthy living environments’. Interview transcripts were coded to identify collaborations and information- and knowledge sharing between stakeholders. Stakeholders were categorized into domains (animal, vector, human, environment, other) and governance levels (international, national, regional, local, other). Networks were visualized and analysed using Cytoscape and R.

**Results:**

Stakeholder analysis identified 87 stakeholders who influence or are (likely to be) influenced by MBV preparedness and response, of whom 47 were identified as having an active role in the MBV interaction network. Network visualisation unveiled 153 connections among these 47 stakeholders, encompassing all domains and governance levels but showed underrepresentation of regional, local and environmental stakeholders. Transcript analysis revealed low urgency for MBVs among these stakeholders as an underlying reason for their underrepresentation in the MBV interaction network. Analysis and visualisation of the networks for the other two themes (“healthy living environment” and “(zoonotic) infectious diseases”) did show multiple connections with environmental and regional/local stakeholders.

**Conclusions:**

The underrepresentation of the environment domain, regional and local stakeholders in the MBV preparedness and response network underlines the remaining challenge of including all relevant stakeholders. We recommend utilising existing collaborations, identified in this study, and central stakeholders to overcome these bottlenecks.

**Supplementary Information:**

The online version contains supplementary material available at 10.1186/s12889-025-21539-4.

## Background

In recent years, Europe has seen the emergence of various mosquito-borne viruses (MBVs) including Zika, Dengue, Usutu, Chikungunya, Sindbis and West Nile Virus (WNV) [1, 2]. In 2023, ECDC reported the highest number of locally acquired cases of dengue (127) in Europe, since the first reports of this disease in 2010, and in 2022, the highest number of WNV cases (1133) since the peak epidemic year in 2018 [1, 3]. With the geographical spread of invasive mosquitoes and the observed increase in MBV emergence in Europe, even more cases and possible deaths of MBVS are expected [1]. This expected increase emphasizes the need for preparedness and response plans for future MBV outbreaks in Europe [1].

Mosquito-borne viruses are a ‘One Health threat’, because their transmission not only relies on the presence of a competent vector species, but also on factors such as susceptible (reservoir) hosts, which can be humans or animals, and suitable environmental conditions (e.g., temperature, precipitation, habitat) [2]. Therefore, all One Health domains should be included in MBV preparedness and response plans. While the relative importance of these domains may vary depending on the stage of disease emergence and disease transmission cycle, their collective involvement in the development of preparedness and response remains crucial. This requires regular sharing of knowledge and information as well as translation of knowledge across domains and governance levels [4–6]. If relevant domains are missing in collaborations, this could lead to delayed or conflicting responses or (unforeseen) problems with preparedness and response implementation [7, 8]. However, while the need for a One Health approach is widely accepted, the involvement of all relevant domains in MBV preparedness and response remains a challenge [4, 7, 9, 10]. There is no pre-defined, universally applicable overview of stakeholders and domains to include in preparedness and response to MBVs, as stakeholder roles and interactions are context specific. Therefore, a first step in preparedness and response planning should be identifying stakeholders’ roles and collaborations, for a certain topic and region, to ensure that all relevant stakeholders, respective domains and governance levels are included [11].

While Southern European countries have experienced various outbreaks of various MBVs in the past decade, Northern European countries are at increasing risk for similar trends in diseases emergence, driven by amongst others climate change, globalization and increases in international trade [1]. Yearly circulation of Usutu since the first detection in 2016, the recent introduction of WNV in Germany and the Netherlands, as well as the recent Blue tongue virus outbreak underpin this [12–16]. The circulation of Usutu and recent introduction of WNV cases in the Netherlands, in combination with an increased detection of invasive mosquitoes such as *Aedes albopictus* and the geographical spread of other MBVs towards Northern-European countries make the Netherlands an interesting case study. Therefore, this study aims to identify stakeholders involved in preparedness and response for MBVs in the Netherlands. Additionally, this study aims to gain insight in collaborations, and knowledge- and information-sharing, between these individual stakeholders, and across their respective domains and governance levels, using social network analysis. This will reveal stakeholders with central positions in the network who could act as brokers in facilitating stakeholder connections. Lastly, we aim to identify underlying causes for bottlenecks in collaborations, information- and knowledge-sharing between stakeholders.

## Methods

### Study design

This study combined a Stakeholder Analysis (SA), allowing for the identification of all relevant stakeholders, with a Social Network Analysis (SNA) which offers a way to measure and evaluate directionality of connections and stakeholder roles within a stakeholder network [17]. We analysed the following interaction types: (1) Information sharing, defined as sharing or receiving data (e.g. signals from surveillance systems, mosquito species abundance) (2) Knowledge sharing or advice (e.g., interpretation of research results, expert knowledge), and (3) Collaborations (e.g., collaborative surveillance, -policy making, -research).

**Fig. 1:**
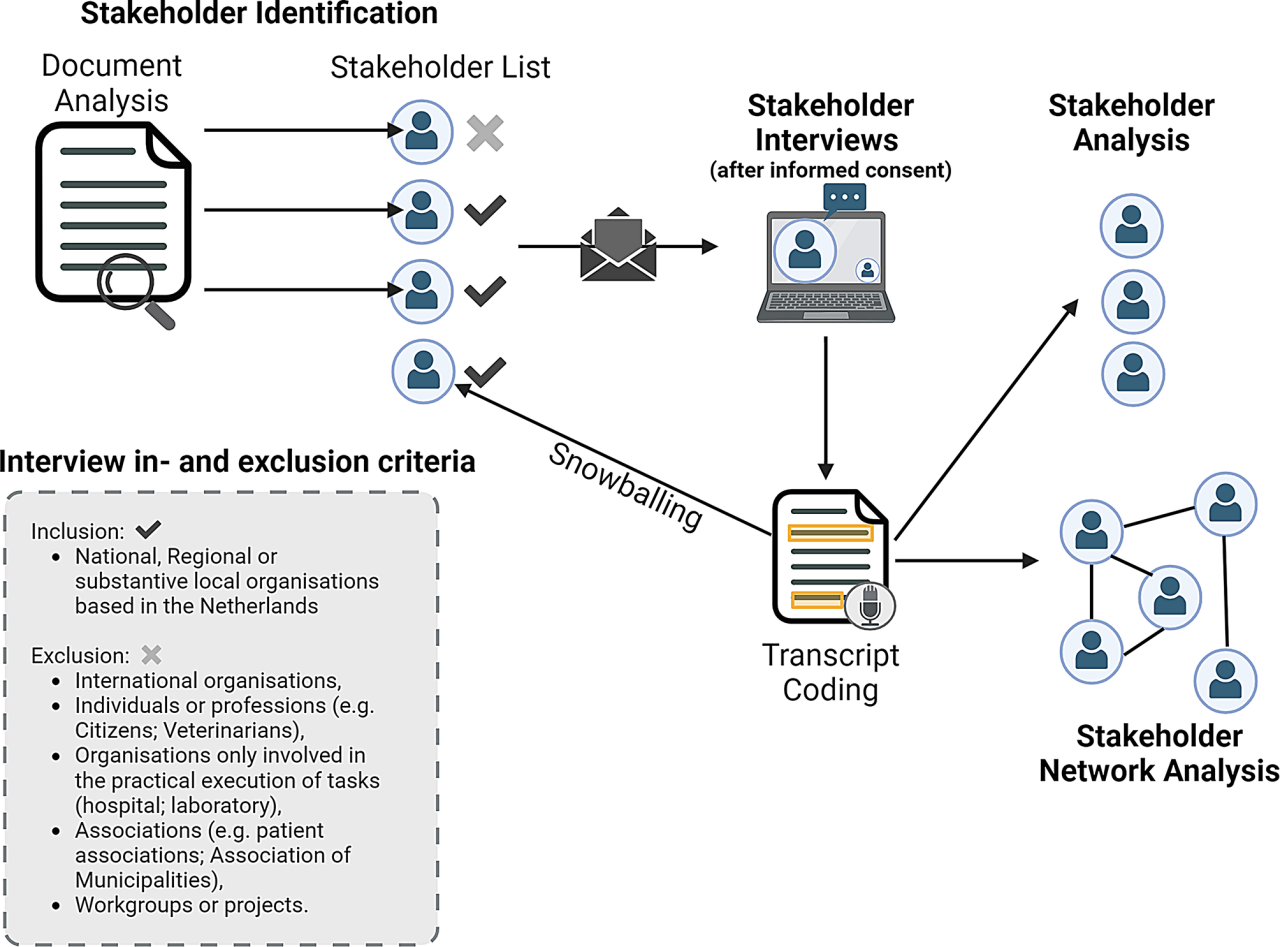
Overview of the study design

### Stakeholder identification

Stakeholder identification was performed through document analysis and interview transcript analysis (snowball sampling), using the following stakeholder definition “all organisations or individuals who influence or are (likely to be) influenced by mosquito-borne virus preparedness and response policy in the Netherlands” (Fig. 1). We applied the OHLEPP “One Health definition” in the stakeholder selection to ensure involvement of all relevant parties [18]. Reports, guidelines and relevant legislation for mosquito or MBV preparedness and response for the Netherlands were identified through a literature search and analysed to identify stakeholders (Additional file 1 A).

### Semi-structured interviews

Due to practical constraints, it was not feasible to conduct interviews with all identified stakeholders. Therefore, stakeholders were selected for interview inclusion based on inclusion and exclusion criteria (Fig. 1). Because of the organizational structure of some stakeholders, multiple sub-organizations (e.g. municipalities) or departments, were invited to ensure a comprehensive understanding of their views. Stakeholders that did not fit the interview inclusion criteria, were still included in the stakeholder overview. The overview of the identified and invited stakeholders, sub-organisations and departments, can be found in additional file 1B.

Permission and informed consent for recording of the interviews, and analysis of the responses, with the aim of publishing a research manuscript, was obtained from each participant at the start of the interview. The interviews were conducted and recorded via Microsoft teams and lasted between 20 and 90 minutes. Two rounds of interviews were held, the first round between June 2021 and June 2022, the second round between January 2023 and July 2023 with a slightly shortened interview guide (Additional file 1 C). Prior to the interview, interviewees received information on the study aims and interview topics, afterwards transcriptions were sent to interviewees for validation. The interview guide included questions about stakeholders’ general responsibilities and their role in MBV preparedness and response; one question on uncertainties and information needs; and one question on their perspectives on the coordination and organisational challenges of MBV preparedness and response (Additional file 1 C). To obtain data on stakeholders’ networks, interviewees were asked 1) on which stakeholder(s) they depend for information, and knowledge or advice related to mosquitoes or MBVs (if any), 2) which stakeholder(s) depend on them for information, and knowledge or advice related to mosquitoes or MBVs (if any), and 3) with which stakeholders(s) they collaborate related to mosquitoes or MBVs. Interviewees were also asked to list any workgroups, knowledge sharing platforms or projects they were involved in related to the topic. Interviewees who indicated they did not have an active role in MBV policy currently, were provided the opportunity to answer questions based on their experiences related to “(zoonotic) infectious diseases” or “healthy living environment and climate adaptation” (from hereon called healthy living environment). Their views and roles for these subjects could still be relevant for this study and help identify stakeholder connections.

### Data analysis

#### Transcript coding

Transcripts were analysed and coded using MAXQDA (Plus 2022 Release 22.5.0) [19]. Coding was performed in two rounds by PdB. First, all transcripts were analysed to identify connections between stakeholders. When a connection was mentioned, a code (name of the connected stakeholder) was assigned to the quote. This first round of coding resulted in a codebook of all mentioned stakeholders fitting the stakeholder definition. Then, a second round was conducted to assign all connections to an interaction type (information; knowledge/advice; collaboration) and connection theme (Mosquito-borne viruses; (Zoonotic) Infectious diseases; Healthy living environment). A distinction was made between current connections and connections in case of “scale-up” e.g. “We don’t need any information on MBV now, but if there is an increase in invasive mosquitoes or if there are MBV cases, we would ask stakeholder X.” Additionally, the direction of connections was coded so directed network plots could be created. All mentioned connections fitting the three interaction types were included, connections did not have to be confirmed by the ‘connected stakeholder’. Additionally, stakeholders’ roles, uncertainties and information needs, and other noteworthy quotes were coded during the second coding round.

Five transcripts were double-coded by a second reviewer (HB), discrepancies in coding between authors were resolved through consensus. Regular meetings to discuss uncertainties in the coding were held throughout the rest of the coding process.

#### Data cleaning and analysis

The final coded quotes were extracted from MAXQDA and imported in Microsoft Excel. In Excel a network dataset was created with the following columns: Stakeholder 1 (interviewee); Stakeholder 2 (interacting stakeholder); Connection theme; Interaction type; Connection direction; “scale up” yes/no; Quote. Each row contained one connection. Data was pseudonymized and analysed on organisation level.

R (version 4.2.2, by RStudio version 2022.) was used for data cleaning of the connection dataset. Data cleaning included grouping stakeholders into domains based on their main roles: human domain (focus on human health), animal (focus on animal health), vector (focus on vectors), environment (focus on environment, including water and climate adaptation), human-animal (focus on both human and animal health), and other (stakeholders with no clear domain (e.g. citizens)) [20]. The human-animal domain was created so stakeholders focussing on both human and animal health, could be assigned into a single domain, this was done to avoid double connections. However, while this domain is plotted separately, the results for this domain should be interpreted as part of both the animal and human domain. Stakeholder organisations with departments operating in different domains (e.g. the health and environment departments of municipalities) were included as separate stakeholders, so the correct domain could be assigned. Additionally stakeholders were grouped into governance levels: International (Stakeholders operating from another nation; cross-border organisations or global organisations e.g. WHO); National (stakeholders operating in the Netherlands at the national level); Regional (stakeholders operating in a specific region of the Netherlands e.g. one or multiple provinces); Local (stakeholder operating in a specific location e.g. municipality); and Other (Stakeholders without a clear governance level e.g. private companies). An overview of how each identified stakeholder was grouped into their respective domain and governance level can be found in additional file 1B.

Cytoscape was used for the network visualisation [21]. Plots were created for each interaction type and connection theme, directed networks were created for information and knowledge/advice sharing between stakeholders. Cytoscape and R (igraph software package) were used to analyse the following Network characteristics: Network density (range 0–1) the connectedness of a network, indicating how many connections exist, relative to the total possible connections; Average number of neighbours, the number of stakeholders that a stakeholder in the network is on average directly connected to; and characteristic path length, the average number of steps needed for a stakeholder to reach another stakeholder in the network [22, 23]. Stakeholders’ positions within each network plot were assessed by calculating closeness- and betweenness centrality (formulas in additional file 1D). Closeness centrality (range 0–1) measures the number of connections between one stakeholder to reach all other stakeholders in the network, reflecting how quickly for example, information, can spread from a stakeholder to all others. Stakeholders with a closeness centrality close to 1 can share information to all other stakeholders in the network with minimal intermediary steps. Betweenness centrality measures how often a stakeholder is the shortest way to connect two other unconnected stakeholders in the network. The range of betweenness centrality can differ greatly depending on the size of the network, therefore scores were normalized (range 0–1) to allow for easier interpretation. Stakeholders with a high betweenness centrality are the shortest path between multiple otherwise unconnected stakeholders and might act as intermediaries in transferring e.g. information between these less connected parts of the network. All stakeholder centrality values are interpreted in relative comparisons within the same network. Since the visualisation of the “(Zoonotic) Infectious diseases” and “Healthy living environment” networks were not the main aim of this paper, these networks were too incomplete to calculate network characteristics and meaningful centrality scores.

## Results

### Stakeholder overview

#### Stakeholder identification

**Fig. 2:**
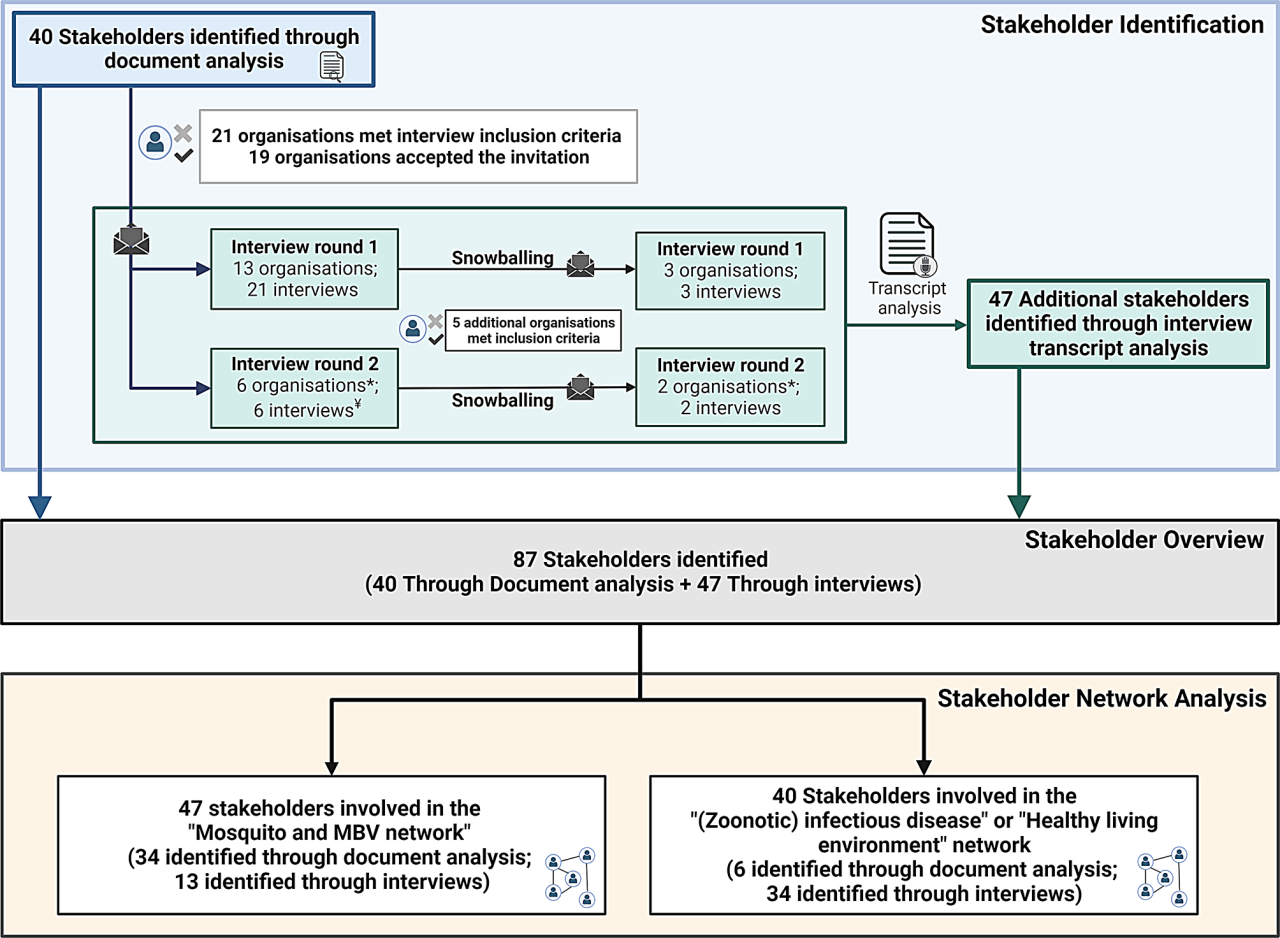
Stakeholder overview: Stakeholder identification (document analysis and interviews), Stakeholder analysis, and Stakeholder network analysis. *An additional department or suborganisation of an already identified stakeholder organisation was also interviewed. ¥ One person was interviewed about their work in two organisations

Document and legislation analysis resulted in the identification of 40 stakeholders fitting the stakeholder definition (Fig. 2). After applying the inclusion and exclusion criteria for interview participation (Fig. 1) 21 stakeholder organisations were eligible and consequently invited. 19 stakeholder organisations accepted the invite, of those 13 were interviewed in the first interview round and six in the second round. These interviews revealed five additional stakeholders (three organisations and two additional departments of already included organisations) who were deemed essential for data collection and were consequently invited for an interview (Fig. 2). In total, 22 organisations were interviewed and, because multiple sub-organisations and departments were included, 32 interviews were held. Analysis of all interview transcripts, to identify stakeholders and stakeholder connections, showed all stakeholders identified through document analysis (40) were mentioned in the interview transcripts. Furthermore, 47 additional stakeholders fitting the stakeholder definition were mentioned in the interview transcript. Together, document analysis and interview transcripts identified 87 stakeholders. A more detailed overview of stakeholders identified, invited and interviewed can be found in additional file 1B.

While all 87 stakeholders were mentioned during the interviews and fit the stakeholder definition, coding and analysis of the interview transcripts showed that not all 87 stakeholders currently have connections in the MBV network. Only 47 stakeholders had an active role in this network, most of these stakeholders were already identified through document analysis but thirteen additional stakeholders were identified through the interviews. The other 40 stakeholders do not currently have an active role in MBV preparedness and response but are involved in either one or both of the other themes ((Zoonotic) infectious diseases; Healthy living environment) (Fig. 2). This included six stakeholders for whom a role in MBV preparedness and response was identified in document analysis which indicates they have a role according to guidelines, reports or legislation, but do not perform these tasks yet.

### Network analysis MBV preparedness and response

**Fig. 3:**
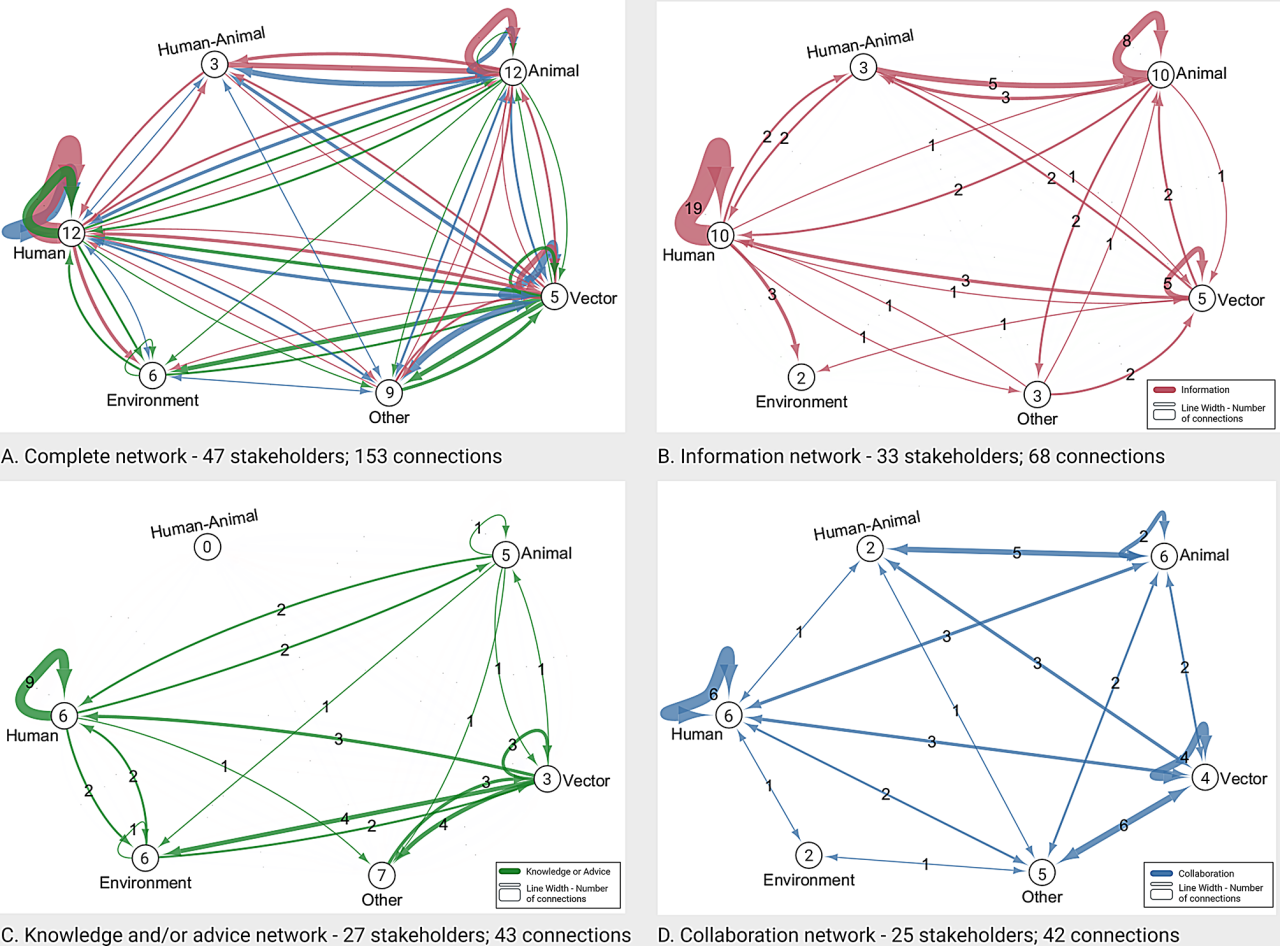
“Mosquito and Mosquito-borne virus” interaction networks between the domains. Panel A shows the complete network with all connections between the different domains (human, animal, human-animal, vector, environment and ‘other’). Panel B. Shows information sharing (in pink). Panel C. shows knowledge and or advice sharing (in green). Panel D. shows collaboration

**Fig. 4:**
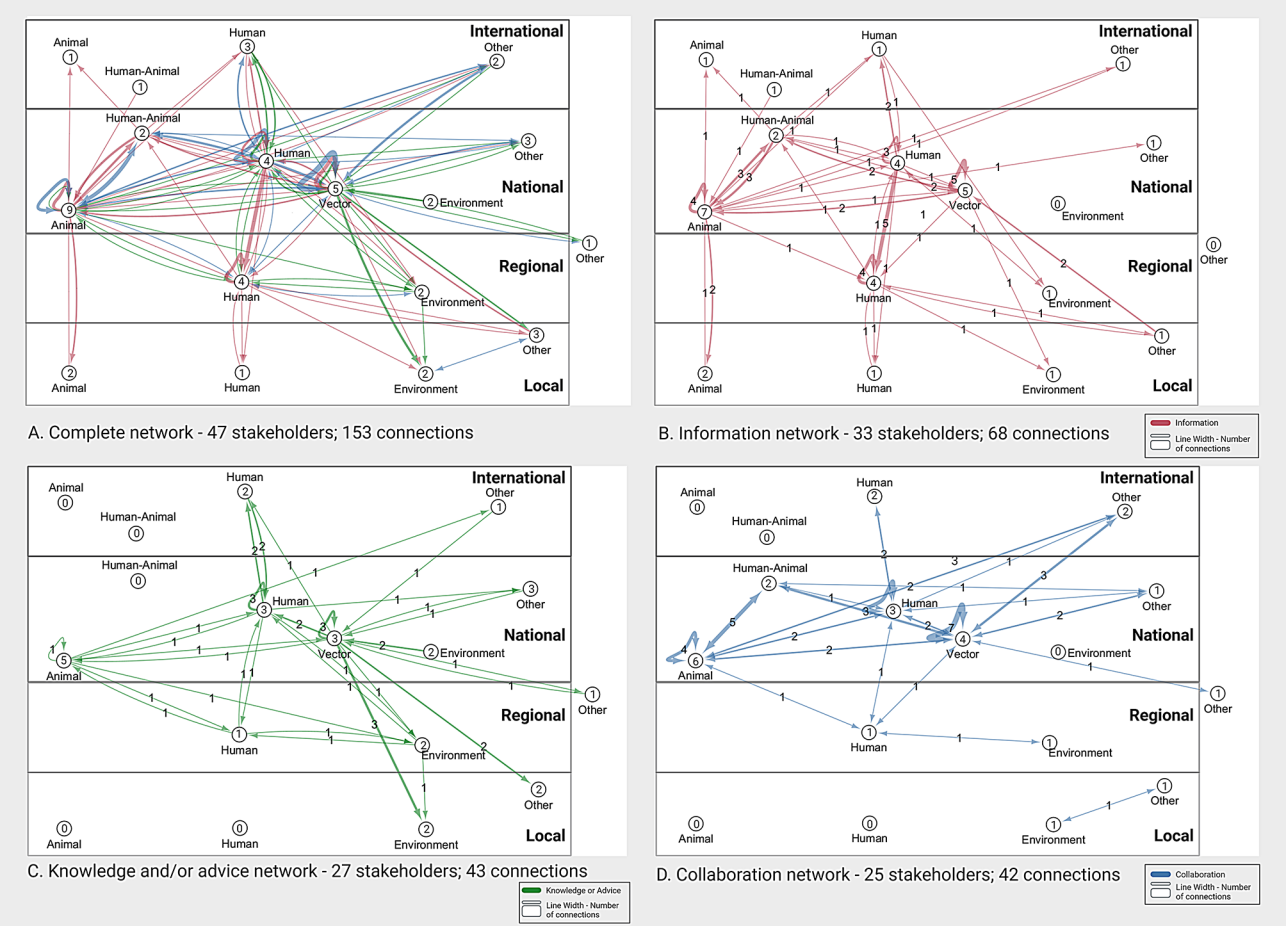
“Mosquito and Mosquito-borne virus” interaction networks between the domains and their respective governance levels. Panel A shows the complete network with all connections between the different domains (human, animal, human-animal, vector, environment and ‘other’) and governance levels (international, national, regional, local, other). Panel B. Shows information sharing (in pink). Panel C. shows knowledge and advice exchange (in green). Panel D. shows collaborations (in blue). Line numbers correspond with the number of connections between domains. Circle numbers correspond with the number of involved stakeholders in each governance level of the domains

During the interviews a total of 153 connections between 47 stakeholders were identified for the network around MBV preparedness and response. These connections were split into the different interaction types: information (33 stakeholders; 68 connections), knowledge and advice (27 stakeholders; 43 connections), and collaborations (25 stakeholders; 42 connections) which shows that not every stakeholder is involved in all three interaction types (Figs. 3A-D and 4A-D). The networks for each interaction type are discussed below.

#### Information sharing network

The MBV information sharing network consists of one network with 68 connections between 33 stakeholders (Fig. 3B, detailed figure in additional file 1E). Stakeholders were on average directly connected to 3.21 other stakeholders (Average number of neighbours) and 2.96 steps were needed to reach another stakeholder in the network (Characteristic path length). The network density is 0.063 (range 0–1), indicating that overall, stakeholders were not strongly interconnected and that multiple steps were needed to reach all stakeholders in the network.

Information was shared between all different domains, but connections were sparser with the environment domain (Fig. 3B). Additionally, the environment domain only receives information on the topic of MBV. Within-domain information exchange was observed for the human domain and in the vector and animal domain but less frequently. Information was mostly exchanged between the national organisations in each domain (Fig. 4B). Information dissemination to international organisations mostly occurred through national organisations. Direct connections between the national and local organisations were present for the animal and vector domain but for the human domain most information was disseminated from national to local level organisations via regional organisations.

#### Knowledge and/or advice sharing network

The knowledge and/or advice network (from here on knowledge/advice) consists of three subnetworks, two isolated networks with connections between only two stakeholders (namely between blood bank and international blood banks and between two national animal health organisations) and one main network with 40 connections between 23 stakeholders (Fig. 3C, detailed figure in additional file 1E). In the main knowledge/advice network, stakeholders were on average directly connected to 2.5 stakeholders and 2.15 steps were needed to reach another stakeholder in the network. The network density is 0.073 (range 0 − 1). This indicates that stakeholders were not strongly interconnected and that multiple steps were needed to get knowledge/advice to all stakeholders in the network.

Knowledge/advice was mostly shared by the vector domain and received by the environment domain (Fig. 3C). Quotes corresponding with these connections showed they were related to advice about mosquito nuisance concerns from citizens in relation to water management and climate adaptation, rather than mosquito-borne virus risks. Limited sharing of knowledge/advice to international and local organisations was observed, except for the vector domain who shared with all different policy levels and had multiple interactions with local stakeholders from the environment and other domain (Fig. 4C). No connections were identified for the human-animal domain, however as described in the methods, the result for this domain should be interpreted as part of both the animal and human domain for which knowledge/advice sharing connections were observed.

#### Collaboration network

The collaboration network consists of two subnetworks, one isolated network between a local environment stakeholder (Municipality [Environment]) and a local ‘other’ stakeholder (Resident association) and one network with 40 bidirectional connections between 23 stakeholders (Fig. 3D, detailed figure in additional file 1E). In the main network, stakeholders were on average directly connected to 3.39 stakeholders and need 2.55 steps to reach another stakeholder in the network. Network density was 0.154 (range 0–1), this indicates stakeholders are interconnected but that multiple steps are still needed to initiate collaborations with all stakeholders in the network.

In the main network, multiple collaborations were present between all domains except for the environment domain, where only one collaboration between a regional human and a regional environmental stakeholder was observed (Figs. 3D and 4D). Collaborations mainly existed between national organisations of the different domains. Few collaborations with international and regional organisations were identified and no collaborations with local organisations. In the isolated network a collaboration on the local level was observed, this is a collaboration between an environment domain stakeholder and an ‘other’ domain stakeholder, showing there are collaborations on this theme. The lack of connection between this isolated network and the main network indicates a bottleneck in collaborations with local stakeholders as well as environmental stakeholders.

#### Connections in case of “scale-up”

**Fig. 5:**
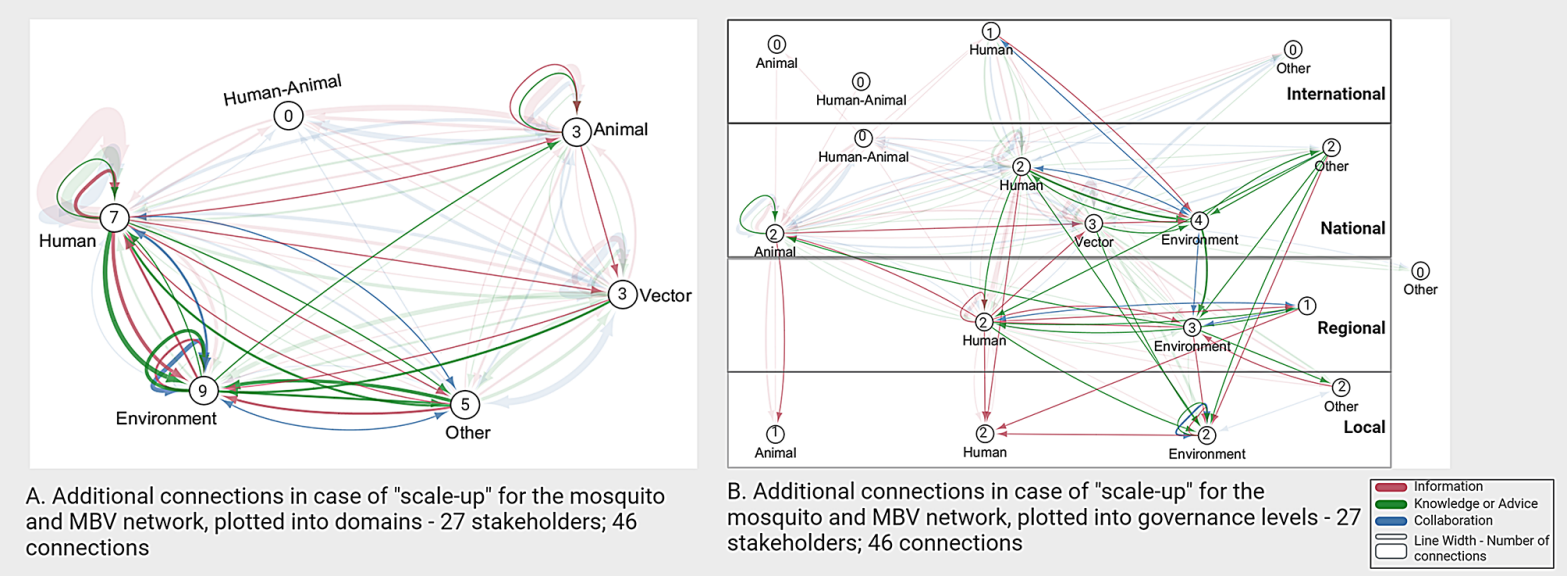
“Mosquito and Mosquito-borne virus” and “scale-up” interaction networks between the domains and their respective governance levels. Panel A shows the complete network with all connections between the different domains (human, animal, human-animal, vector, environment and ‘other’). Panel B. shows the inter governance level (international, national, regional, local, other) connections. Circle numbers correspond with the number of involved stakeholders in each domain and respective governance level. Darker lines show the additional connections in case of “scale-up”, lighter lines show the original “Mosquito and Mosquito-borne virus” network connections

In addition to the current connections, Fig. 5 shows the connections in case of “scale-up”. This network mainly shows additional connections between the human and environment domains on the national, regional and local level. Most “scale-up” connections are either information or knowledge sharing.

### Central stakeholders and their roles

Tables 1 and 2 show the stakeholder organisations with the highest closeness- and betweenness centrality for each network plot. Stakeholders with a high closeness centrality can share with all other stakeholders in the network with minimal intermediary steps. Stakeholders with a high betweenness centrality are the shortest path between multiple otherwise unconnected stakeholders and might act as intermediaries between these less connected parts of the network. Centrality results for all stakeholders can be found in additional file 1E.

**Table 1:** Top 5 highest scoring stakeholders on closeness centrality (range 0–1)

Domain and governance level	Name of the organisation	Closeness centrality
**Information Network**		
Vector National	National Centre Vector Monitoring	0.4902
Human-Animal National	Erasmus Medical Center (Arbovirus reference laboratory)	0.4310
Human National	National Public Health Institute [ID]	0.4237
Vector National	University (Wageningen)	0.4098
Human Regional	Municipal (public) health service [ID]	0.3906
**Knowledge and/ or advice Network**		
Human National	National Public Health Institute [ID]	0.7778
Vector National	National Centre Vector Monitoring	0.6364
Environment Regional	Municipal (public) health service [Environment]	0.5833
Animal National	Food and Consumer Product Safety Authority	0.5833
Human Regional	Municipal (public) health service [ID]	0.5833
**Collaboration Network**		
Vector National	University (Wageningen)	0.5641
Human National	National Public Health Institute [ID]	0.5641
Vector National	National Centre Vector Monitoring	0.5500
Other International	International Partners	0.5116
Animal National	National Wildlife health centre	0.4681

[ID] = Infectious diseases

Central stakeholders in the information sharing network were stakeholders who have an active role in the surveillance of MBVs, perform research on mosquitoes and MBVs or were responsible for the regional response to MBVs (Tables 1 and 2). Stakeholders with an overall low centrality were stakeholders involved in task execution (hospitals; microbiology labs) or stakeholders in the environment domain.

Stakeholders with high closeness centrality in the knowledge/advice sharing network generally have an active role in policy making for MBVs or are stakeholder organisations with an advisory or executive role towards local organisations (mostly municipalities) (Table 1). Values for betweenness centrality differ greatly and only three stakeholders, of whom two from the vector domain, showed a high betweenness centrality (Table 2). These stakeholders have an active role in either policy making for MBVs or invasive mosquito monitoring and control. Stakeholders with low centrality were mostly environment and ‘other’ local organisations who only receive knowledge. Stakeholders with a moderate closeness but low betweenness centrality were stakeholders who only shared knowledge.

**Table 2:** Top 5* highest scoring stakeholders on normalized betweenness centrality (range 0–1)

Domain and governance level	Name of the organisation	*N* Betweenness Centrality
**Information network**		
Human National	National Public Health Institute [ID]	0.1664
Human-Animal National	Erasmus Medical Center (Arbovirus reference laboratory)	0.1488
Human Regional	Municipal (public) health service [ID]	0.1337
Vector National	University (Wageningen)	0.0953
Vector National	National Centre Vector Monitoring	0.0842
**Knowledge and/ or advice Network**		
Vector National	National Centre Vector Monitoring	0.1123
Human National	National Public Health Institute [ID]	0.0531
Vector National	Pest Control Company	0.0508
**Collaboration Network**		
Vector National	National Centre Vector Monitoring	0.2281
Vector National	University (Wageningen)	0.2239
Human National	National Public Health Institute [ID]	0.1928
Other International	International Partners	0.1721
Human National	Ministry of health	0.1540

*Values below 0.05 were not included in the table, which could mean less than 5 stakeholders are shown. [ID] = Infectious diseases. N = Normalized

Stakeholders with high closeness centrality in the collaboration network have an important role in (inter)national research on mosquitoes and MBV’s, the other stakeholders have a role in the animal, vector and human surveillance and/or policy making for MBVs (Table 1). The same stakeholders have a high betweenness centrality except for the national animal health organisation (National Wildlife health centre), instead a national human health organisation (Ministry of Health) has a high betweenness centrality (Table 2). This organisation has an important role in policy making for MBVs. All stakeholders in the network had moderately high closeness centrality scores, betweenness centrality scores showed more variety.

Overall, local and regional organisations in all domains, and environment domain stakeholders in general are underrepresented in all networks and have lower centrality scores.

### Stakeholder interview perspectives

Transcript analysis identified several underlying reasons for the underrepresentation of environmental and local or regional stakeholders. Urgency and recognition of potential problems arising from mosquitoes was absent or low among stakeholders from the environment domain. Additionally, participants mentioned “living environment” and “public health” are often tackled as separate task, allocated to different organisations or departments. Participants described that, various organisational differences, such as jargon, complicate knowledge and information exchange as well as collaborations. However, it was also noted by regional and local stakeholders that increased efforts are undertaken to integrate tasks between the environment and human domain. Environmental stakeholders also mentioned a potential conflict in priorities, they questioned whether taking measures to reduce mosquitoes might affect one of their main priorities, biodiversity. If it were necessary to make changes in environmental design to reduce mosquito abundance, they indicated they currently lack the required knowledge to do so and are in need of guidelines.

Potential reasons identified for the underrepresentation of local/regional stakeholders were amongst others, decentralization of tasks to multiple regional and local organisations, leading to a high number of stakeholders and fragmented approach which is subject to regional differences. The fragmentation of tasks and responsibilities between different organisations and respective departments was noted to lead to a lack of clarity on whom to contact or involve. Furthermore, a low sense of awareness for MBVs, among local actors such as veterinarians and general practitioners was mentioned. While participants acknowledged that this was not unexpected, since vector-borne diseases are only a fragment of their responsibilities, it was a concern of multiple participants as it might lead to a delay in the early warning of mosquito-borne diseases.

### Network analysis for related themes

**Fig. 6:**
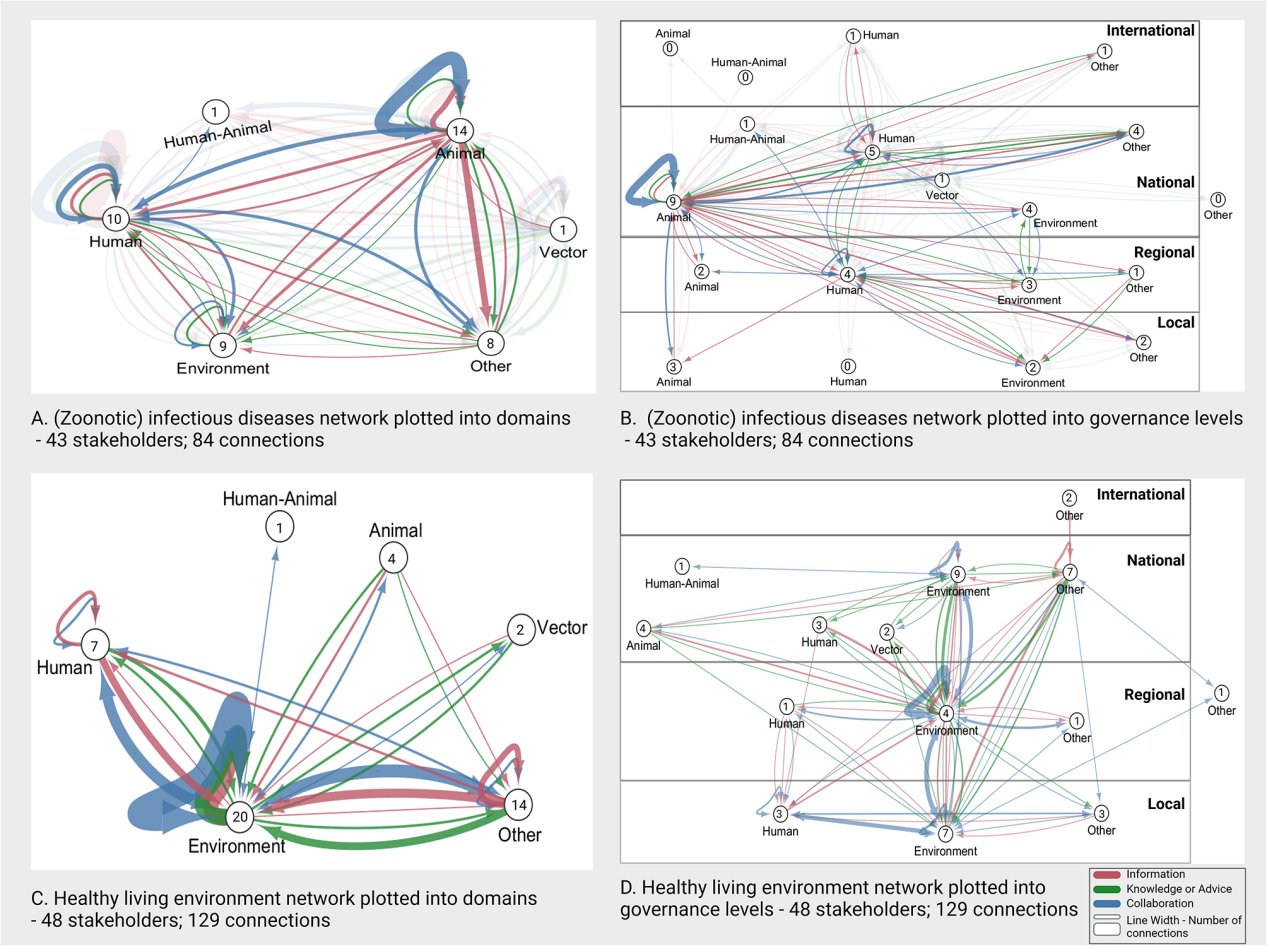
“(Zoonotic) infectious diseases” and “Healthy living environment” interaction networks between the domains and their respective governance levels. Panel A shows the complete network with all connections between the different domains (human, animal, human-animal, vector, environment and ‘other’) for the “(Zoonotic) infectious disease” network. Panel B. shows the inter governance level (international, national, regional, local, other) connections for the “(Zoonotic) infectious diseases” network. Panel C. shows the complete network with all connections between the different domains for the “healthy living environment” network. Panel D. shows the inter governance level connections for the “healthy living environment” network. Circle numbers correspond with the number of involved stakeholders in each domain and or governance level

Analysis of connections fitting the other two themes ((Zoonotic) Infectious diseases; Healthy living environment) revealed different network dynamics. The zoonotic disease network overlaps with the MBV network but revealed more connections between the animal domain and the environment domain, between the human domain and environment domain, and in general more connections with regional and local stakeholders (Fig. 6). The additional connections identified for this theme can possibly be utilized for MBV preparedness and response in the future. In the “Healthy living environment network” all domains have connections with the environment domain rather than with each other (Fig. 6). The human domain is most connected to the environment domain, with multiple connections for information and knowledge sharing as well as collaborations. These connections are mostly present on the regional and local level. This network visualisation indicates that the environment domain operates largely at the regional and local level.

## Discussion

This study identified 87 stakeholders who influence or are (likely to be) influenced by MBV preparedness and response. Of those stakeholders only 47 were recognized as currently having an active role in collaborations or information and knowledge/advice sharing related to mosquitoes and MBVs. The networks of interactions between these stakeholders, their respective domains and governance levels showed underrepresentation of the environment domain, as well as of stakeholders from the regional and local governance level. The Human, Animal and Vector domains and the national level were mostly represented in the networks. The 40 remaining stakeholders identified during our study were described to have a role in either or both of the other two networks “(zoonotic) infectious diseases” and “healthy living environment” these networks showed more connections with regional, local and environmental stakeholders.

### One health interactions and collaborations

Visualisation of the MBV network showed substantial lower involvement of the environment domain compared to the other domains, especially for collaborations. Furthermore, for information and knowledge/advice sharing, environmental stakeholders were mainly on the receiving end. The lack of involvement of the environment domain is consistent with previous literature describing the absence of environmental or ecology stakeholders in One Health initiatives, guidelines or publications [24–27]. Potential reasons described in literature, are the difference in values and priorities between actors, and lack of clarity among stakeholders from the other domains about the potential roles of environmental stakeholders, which serve as obstacles in collaboration [24, 26, 27]. In our study, stakeholders from the environment domain mentioned that MBV prevention measures and response might conflict with priorities of their organisations and that MBVs are currently not their priority. The absence of collaborations and the unidirectionality of information and knowledge/advice sharing might contribute to this, since this leaves the potential of their contributions unexplored. However, several studies have described the importance of including (urban) ecology and environmental stakeholders in vector-borne diseases policymaking. For example, Ellwanger et al. state that “an ecological perspective can provide foresight into the appropriateness of interventions, provide answers to unexpected vector control responses, and contribute to effective management solutions in an ever-changing environment” [27]. Therefore, we recommend increasing efforts to include the environment domain. The identified connections in case of “scale-up”, between the human and environment domain are promising and indicate stakeholders are aware of each other. Pre-existing network structures can provide a foundation for effective collaborations and information flow between stakeholders [28]. This study showed a pre-existing network around “healthy living environment” with a central role for the environment domain and interactions with all other domains. Stakeholders and connections identified within this network can be used to include the environment domain in preparedness and response for MBVs.

### Governance level involvement

Previous studies have reported the importance of collaborations between local, regional, national and international governance levels for preparedness and response [4, 28, 29]. However, the visualisation of the MBV networks revealed that not all governance levels are reached for each interaction type, especially in the “collaboration network” local organisations were not involved.

Local stakeholders are reached with information and some with and knowledge/advice. However, this does not inherently translate into subsequent actions by stakeholders. Integration of information and knowledge can be hindered by several factors including a difference between what the sharing organisations mean and what the receiving organisations understand [30]. Engaging stakeholders from all governance levels in decision making and negotiations around preparedness and response improves information and knowledge integration [29]. Furthermore, inclusion of stakeholders at all levels was described as crucial for the implementation of the One Health approach for infectious disease control by Mazet et al. [4]. Fragmentation of tasks, leading to unclarity on whom to involve, was mentioned as one of reasons for the lack of inclusion. Existing connections, as identified in the “(zoonotic) infectious diseases” and “healthy living environment” network, could be utilized to identify and connect with relevant regional and local stakeholders. Additionally, efforts to improve integration of health and environment stakeholders on the regional and local level (such as the Dutch “environment and planning act”) can further support collaborations between regional and local stakeholders [31].

### Stakeholders and central roles

Previous literature distinguished between three types of stakeholders: scientists; public-health decision makers and practitioners; and considered all these stakeholders critical to the effective translation of data to public health emergency prevention, detection, and response [8]. While our results identified central roles for scientists and public health decision/policy maker, practitioners, who are mostly local stakeholders, were not identified as central stakeholders in the current networks around MBV preparedness and response. This indicates that these stakeholders are less connected and multiple intermediary steps are needed to reach these stakeholders. The identified stakeholders with high closeness and betweenness centrality could act as brokers in facilitating connections with these stakeholders. Including these stakeholders when disseminating information or knowledge to less connected stakeholders, or when seeking collaborations, can improve the chances of successful integration.

### European context

While this research focusses on the Netherlands and the involvement of stakeholders is context specific, findings on stakeholder involvement can be translated to other European countries and potentially even non-European countries with a similar level of threat for emerging MBVs. The Dutch public-health preparedness and response structure complies with the International Health regulation (IHR), similar translations of the IHR can be expected for national preparedness and response in other WHO member states [32]. Furthermore, changes in climate and the necessity to adopt climate adaptation measures is not limited to the Netherlands. Therefore, even though the exact composition of stakeholder organisations might differ between countries, similar organisations, functions and contexts will exist for other (European) countries. Nevertheless, situational variances may affect the direct application of this research to different contexts, necessitating careful consideration or repetition of the presented methods.

### Limitations

This study has several limitations. A limitation of the applied methods in this study is the lack of distinction between different types of mosquito-borne viruses, as this could affect the involvement and roles of some of the identified stakeholders. Furthermore, no distinction was made between stakeholder involvement in preparedness and stakeholder involvement in response. While this might have resulted in more tailored results, the current analysis offers more general conclusions. This study focused on specific interactions between stakeholders while other dependencies between stakeholders like finances and legitimacy could influence these connections [33]. Excluding these underlying influences might oversimplify the network between stakeholders. Last of all, the applied methods, semi-structured interviews, offer a less structured and uniform data collection than questionnaires could have. However, interviews give more insight into stakeholder’s perceptions and are more informative for a preliminary evaluation of potential reasons for network bottlenecks.

## Recommendations and conclusions

The current study showed the limited inclusion of the environment domain in the MBV preparedness and response network underlining the remaining challenge of multi-sectoral collaboration. Furthermore, the sparse connections with regional and local stakeholders, especially regarding collaborations, imply that increased efforts are needed to connect with stakeholders from all governance levels. The “(zoonotic) infectious diseases” and “healthy living environment” networks revealed existing collaborations which can be used as steppingstones for creating these connections for MBV preparedness and response. In light of the imminent emergence of mosquito-borne viruses, periodical updates of preparedness and response plans are essential. Therefore, to prevent flaws in the preparedness and response to future MBV outbreaks and threats, we recommend policy makers to increase efforts to include all domains and governance levels through central stakeholders and by utilising existing collaborations.

## Electronic supplementary material

Below is the link to the electronic supplementary material.

Supplementary Material 1

## Data Availability

The datasets generated and analysed during the current study are available from the corresponding author on reasonable request.

## Abbreviations

MBVs: Mosquito-borne viruses
WNV: West Nile Virus
SA: Stakeholder Analysis
SNA: Social Network analysis
OHLEPP: The One Health High Level Expert Panel
ID: Infectious diseases
N: Normalized
IHR: International Health regulation
