# Supplementary material for: One Health preparedness and response for mosquito-borne viruses: a stakeholder- and social network-analysis in the Netherlands
Source: BMC Public Health. 2025 Jan 24;25:307. doi: 10.1186/s12889-025-21539-4 (PMC11758748; doi:10.1186/s12889-025-21539-4)
Supplement: Supplementary file 1 — Supplementary Material 1 [file 12889_2025_21539_MOESM1_ESM.pdf]

Additional file 1

Additional file 1 1

Additional file 1A - Stakeholder identification, document analysis 2

Additional file 1B – Stakeholder overview (Stakeholders: Identified; Invited; Interviewed (round); Domain; Governance level) 4

Additional file 1C - Interview guide(s) 12

Interview guide - Interview round 1. 12

Interview guide - Interview round 2. 13

Additional file 1D– Stakeholder centrality tables 16

Additional file 1E – Detailed stakeholder network figures 22

References: 28

Additional file 1A - Stakeholder identification, document analysis

Stakeholder identification was performed through document analysis and snowball sampling using the following stakeholder definition “all organisations or individuals who affect or are (likely to be) affected by mosquito-borne virus preparedness and response policy in the Netherlands now and in the future”. Document analysis was performed by PdB and HB. Reports and guidelines of organisations involved in mosquitoes and mosquito-borne viruses were analysed to identify stakeholders. Relevant legislation was identified using the Dutch public law database (accessible through wetten.overheid.nl) using the search terms (in Dutch): “mosquito” and “infectious disease”.

The following documents were identified and analysed for stakeholder identification:

- Yvonne Huizer KH AJ, Corien Swaan, Ronald Reekers, Rob van Kessel, Ronald ter Schegget, Reinoud Wolter. General protocol infectious diseases [Generaal draaiboek infectieziekten]. Website National Institute for Public Health and the Environment [RIVM]; 2014 [1].
- National Institute for Public Health and the Environment [RIVM]. International Health Regulations [Internationale Gezondheidsregeling]. Website National Institute for Public Health and the Environment (RIVM); 2009 [2].
- Peter Jacobs RvK, Mauro de Rosa, Ife Slegers - Fitz-James, Charlotte Verbart. Vademecum Zoonoses [Vademecum zoönosen]. National Institute for Public Health and Environment [Rijksinstituut voor Volksgezondheid en Milieu (RIVM)]; 2021 [3].
- Sabine Bantjes, Marieta Braks. Exotic Mosquitoes - Policy for invasive mosquitoes in the Netherlands [Exotische steekmuggen - Beleid bij invasieve exotische steekmuggen in Nederland]. Webpage National Institute for Public Health and the Environment (RIVM); 2021 [4].
- Koopmans MPG. Preparing for vector-borne virus outbreaks in a changing world: a One Health Approach Dutch National Research Agenda, Research along Routes by Consortia, Full proposal form 2018, Version 20181010. (Not published online). Netherlands Organisation for Scientific Research; 2018 [5].

The following legislative documents were identified and analysed for stakeholder identification:

- Public Health Act [Wet publieke gezondheid], (2021) [6].
- International Health Regulations [nternationale Gezondheidsregeling (2005), Genève, 23-05-2005], (2005) [7].
- Municipality Act [Gemeentewet] (2021) [8].
- Animal Health and Welfare Act [Gezondheids- en welzijnswet voor dieren], (2019) [9].
- Environment and Planning Act [Omgevingswet], (In development) [10].

The analysis of these guidelines, reports and legislation together resulted in the identification of 40 stakeholder organisations, through document analysis.

Additional file 1B – Stakeholder overview (Stakeholders: Identified; Invited; Interviewed (round); Domain; Governance level)

Stakeholder identification was performed through document analysis and snowball sampling (transcript analysis) using the following stakeholder definition "all organisations or individuals who influence or are (likely to be) influenced by mosquito-borne virus preparedness and response policy in the Netherlands". A distinction can be made between different types of identified stakeholders: A) Overarching stakeholder groups. B) Stakeholder organisations. C) Stakeholder sub-organisations. D) Stakeholder departments. E) Stakeholder individuals or professions.

Overarching Stakeholder groups: A collective name for multiple organisations. Multiple organisations belonging to these overarching groups can be relevant for the subject and can have different roles or views on the matter. Therefore, various organisations within these groups could be identified as stakeholders and invited for the interviews. However, it is also possible that only the overarching Stakeholder groups are mentioned, or they are mentioned as well as the organisations with these groups. Examples are: Academia and Central Government.

Stakeholder organisations: An organisation identified in document analysis or in the interviews as being relevant for the subject because of its role(s) or views.

Stakeholder sub-organisations: An organisation which has multiple regional or local sub-organisations which operate individually. The sub-organisations have the same or very similar roles and can be seen as one main stakeholder organisation, however differences in their regions might influence their roles and views. Therefore, multiple sub-organisations are included in the study. Examples are: Municipalities (345 in the Netherlands), Provinces (12 in the Netherlands), Municipal (public) health organisations (25 in the Netherlands), Dutch Water Boards (21 in the Netherlands).

Stakeholder departments: Stakeholder organisations with multiple departments, which have different roles or views on the subject or belong to different domains. When these departments are named as separate stakeholders within an organisation, they are included separately in stakeholder overview and the network analysis. Examples are Municipality health department and Municipality Environmental departments; National Public health institute (infectious diseases) and National Public Health institute (Environment).

Stakeholder individuals or professions: Stakeholders who do not belong to one organisation and are rather individual stakeholders or a profession. Examples of stakeholders belonging to this category are Citizens; Veterinarian(s); Private Companies and Bird Ringers.

An overview of the different stakeholder types identified, interviewed and mapped in the network plots is presented in figure 1. An overview of the identified and invited stakeholders, stakeholder organisations, sub-organisations and departments, can be found in table 1.

Figure 1. Expanded version Stakeholder identification, interviews and network involvement.

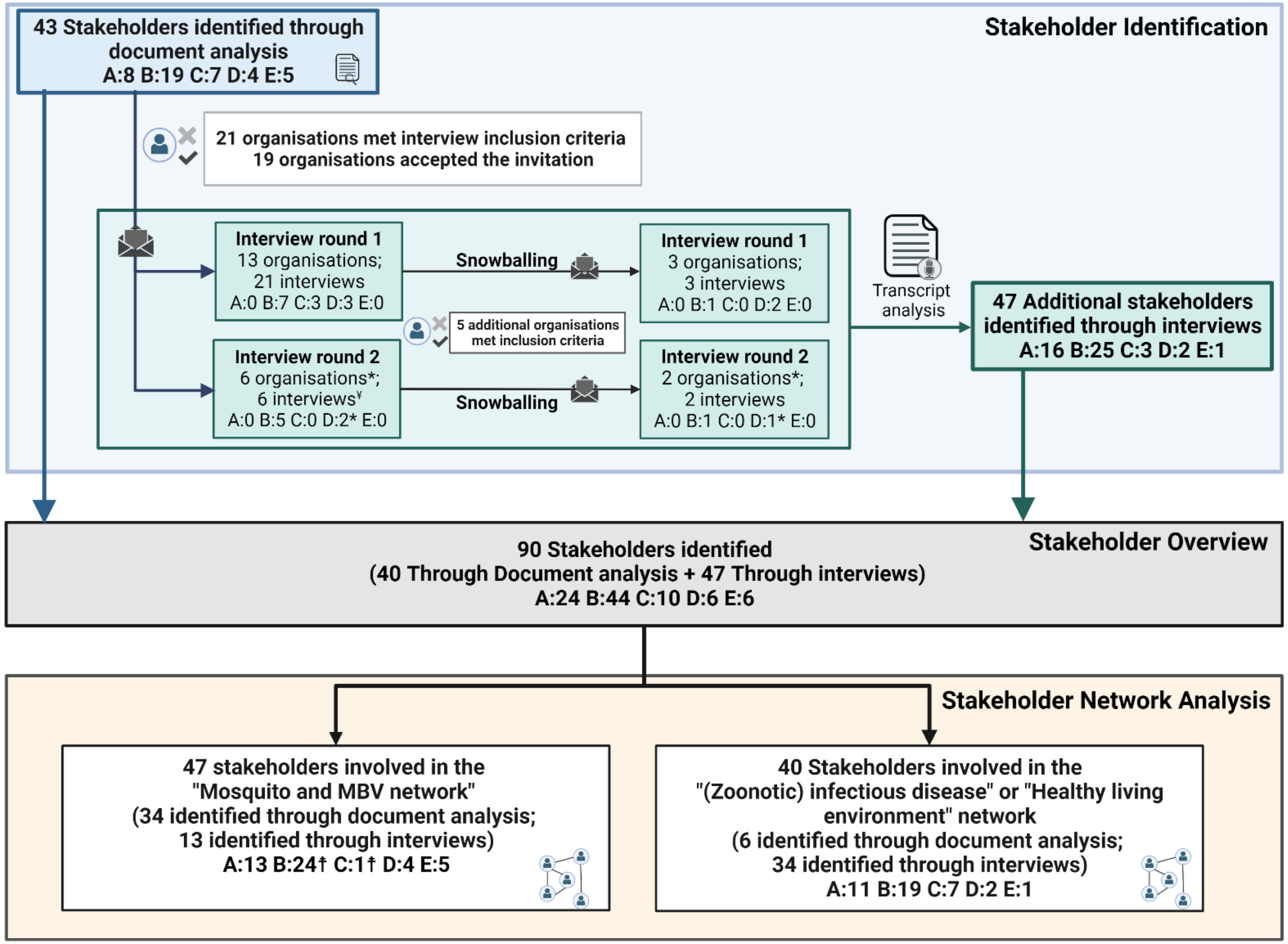

A) Overarching stakeholder groups. B) Stakeholder organisations. C) Stakeholder sub-organisations. D) Stakeholder departments. E) Stakeholder individuals or professions.

* An additional department (D) was interviewed of an already identified stakeholder organisation. ¥ One person was interviewed about their role in two organisations. †

Three organisation (B:1, C:2) were identified as stakeholders, but only specific departments of these organisations were mentioned for the network connections, therefore these organisation names do not appear in the network plots.

Table 1. Overview of the identified stakeholders.

Stakeholder (organisation) names	Type	Identified through:	Invited for an interview	Interview Round	Number of sub-organisations/ departments interviewed	Number of interviewees	Domain	Governance level	MBV network
**Academia**	**A**	Document analysis	No	-	-	-	Other	National	Yes
University (Leiden)	**B**	Document analysis	Yes	-	-	0	Vector	National	Yes
University (Rotterdam)	**B**	Document analysis	Yes	2	1	1*	Human-Animal	National	Yes
University (Utrecht)	**B**	Document analysis	No	-	-	0	Animal	National	Yes
University (Wageningen)	**B**	Document analysis	Yes	1	1	2	Vector	National	Yes
Academic Collaborative Centre	**C**	Document analysis	No	-	-	0	Human	Regional	No
Academic Hospitals	**C**	Document analysis	Yes	-	-	0	Human	Regional	No
Agriculture sector	**A**	Document analysis	No	-	-	0	Environment	Local	No
Animal Shelters	**C**	Snowballing	No	-	-	0	Animal	Regional	No
Animal health service (GD)	**B**	Document analysis	Yes	2	1	1	Animal	National	Yes
Association of public health and safety services	**B**	Document analysis	No	-	-	0	Human	National	No
Association of Water Authorities	**B**	Snowballing	No	-	-	0	Environment	National	No
Bioveterinary Research (laboratory)	**B**	Document analysis	Yes	2	1	1	Animal	National	Yes
Bird Ringers	**E**	Document analysis	No	-	-	0	Animal	Local	Yes
Blood Bank	**B**	Document analysis	Yes	2	1	1	Human	National	Yes

**Central Government**	**A**	Document analysis	No	-	-	-	Other	National	Yes
Ministry Agriculture and Nature	**B**	Document analysis	Yes	1	1	1	Other	National	Yes
Ministry Economics and Climate	**B**	Snowballing	No	-	-	0	Environment	National	No
Ministry of Health	**B**	Document analysis	Yes	1	1	2	Human	National	Yes
Citizens	**E**	Document analysis	No	-	-	0	Other	Local	Yes
Civil Society Organisations	**A**	Snowballing	No	-	-	0	Human	Local	No
Consulting Companies	**A**	Snowballing	No	-	-	0	Other	National	No
Database (Flora and Fauna)	**B**	Snowballing	No	-	-	0	Other	National	No
Database (National Statistics)	**B**	Snowballing	No	-	-	0	Other	National	No
Delta Commissioner	**E**	Snowballing	No	-	-	0	Environment	National	No
Dutch Veterinary Association	**B**	Snowballing	No	-	-	0	Animal	National	Yes
Dutch Water Authorities	**C**	Document analysis	Yes	1	3	3	Environment	Regional	Yes
EASIN (EU, Alien Species)	**B**	Snowballing	No	-	-	0	Other	International	No
ECDC	**B**	Document analysis	No	-	-	0	Human	International	Yes
EEA (European Environmental Agency)	**B**	Snowballing	No	-	-	0	Environment	International	No
Environment Agencies	**C**	Snowballing	No	-	-	0	Environment	Regional	No
Environmental Assessment Agency	**B**	Snowballing	Yes	1	1	1	Environment	National	No
Environmental Developers	**A**	Snowballing	No	-	-	0	Environment	Local	No
Erasmus Medical Center (Arbovirus reference laboratory)	**B**	Document analysis	Yes	2	1	1*	Human-Animal	National	Yes
European Union	**A**	Snowballing	No	-	-	0	Other	International	No
Food and Consumer Product Safety Authority	**B**	Document analysis	Yes	1	1	1	Animal	National	Yes

General Practitioner(s)	**E**	Document analysis	No	-	-	0	Human	Local	Yes
Health Organisations	**A**	Snowballing	No	-	-	0	Human	Local	No
Horse Sector Association	**B**	Snowballing	No	-	-	0	Animal	National	Yes
Hospitals	**A**	Document analysis	No	-	-	0	Human	Regional	Yes
Housing Corporation(s)	**A**	Snowballing	No	-	-	0	Environment	Local	Yes
Hunter Organisation	**B**	Snowballing	No	-	-	0	Animal	Local	No
Infrastructure and Water Management	**B**	Snowballing	No	-	-	0	Environment	National	No
International Blood Bank(s)	**A**	Snowballing	No	-	-	0	Human	International	Yes
International Governments	**A**	Document analysis	No	-	-	0	Other	International	Yes
International Partners	**A**	Snowballing	No	-	-	0	Other	International	Yes
Knowledge institute(s)	**A**	Snowballing	No	-	-	0	Other	National	No
Knowledge Institute (Water, Subsurface)	**B**	Snowballing	No	-	-	0	Environment	National	Yes
Knowledge Institute (Insects)	**B**	Snowballing	No	-	-	0	Vector	National	No
Knowledge Institute (Sea Research)	**B**	Snowballing	No	-	-	0	Animal	National	No
Knowledge Institute (Bird Research)	**B**	Snowballing	No	-	-	0	Animal	National	Yes
Knowledge Institute (Water Authorities)	**B**	Snowballing	No	-	-	0	Environment	National	No
Land Management Organisations	**A**	Snowballing	No	-	-	0	Environment	Local	No
Local Enterprise(s)	**A**	Snowballing	No	-	-	0	Other	Local	Yes
Mammal Association	**B**	Snowballing	No	-	-	0	Animal	National	No
Microbiology labs	**A**	Document analysis	No	-	-	0	Human	Regional	Yes
**Municipalities**	**C**	Document analysis	Yes	-	-	-			
Municipality [Environment]	**D**	Document analysis	Yes	1	3	3	Environment	Local	Yes
Municipality [Health]	**D**	Document analysis	Yes	2	1	1	Human	Local	No

**Municipal (public) health service**	**C**	Document analysis	Yes	-	-	-			
Municipal (public) health service [ID]	**D**	Document analysis	Yes	1 and 2	4	5	Human	Regional	Yes
Municipal (public) health service [Environment]	**D**	Snowballing	Yes	1 and 2	2	2	Environment	Regional	Yes
**National Public Health Institute**	**B**	Document analysis	Yes	-	-	-			
National Public Health Institute [ID]	**D**	Document analysis	Yes	1	2	2	Human	National	Yes
National Public Health Institute [Environment]	**D**	Snowballing	Yes	1	1	2	Environment	National	No
National Centre Vector Monitoring	**B**	Document analysis	Yes	1	1	2	Vector	National	Yes
National Wildlife Health Centre	**B**	Document analysis	Yes	1	1	1	Animal	National	Yes
Nature Organisation(s)	**A**	Snowballing	No	-	-	0	Environment	National	No
Parks Department	**C**	Snowballing	No	-	-	0	Environment	Local	No
Patient Organisation(s)	**A**	Document analysis	No	-	-	0	Human	National	Yes
Pest Control Company	**B**	Document analysis	Yes	1	1	1	Vector	National	Yes
Platform Invasive Species	**B**	Snowballing	No	-	-	0	Vector	National	Yes
Port (Rotterdam)	**B**	Snowballing	No	-	-	0	Environment	Regional	No
Private Companies	**A**	Snowballing	No	-	-	0	Other	Other	Yes
Project Developers	**A**	Snowballing	No	-	-	0	Environment	Local	No
Province(s)	**C**	Document analysis	Yes	1	1	2	Environment	Regional	No
Reference laboratories	**A**	Document analysis	No	-	-	0	Human-Animal	International	Yes
Research Institute (Living Environment)	**B**	Snowballing	No	-	-	0	Environment	National	No
Research Institute (Meteorology)	**B**	Snowballing	No	-	-	0	Environment	National	Yes
Research institute (Human Demographics)	**B**	Snowballing	No	-	-	0	Environment	National	No

Research Institute (Ecology and Birds)	**B**	Snowballing	Yes	2	1	1	Animal	National	Yes
Research Institute (Biology and Environment)	**B**	Snowballing	No	-	-	0	Environment	National	No
Resident Association(s)	**A**	Snowballing	No	-	-	0	Other	Local	Yes
Safety and Crisis Management	**C**	Document analysis	Yes	1	1	2	Other	Regional	No
Secondary Care Physicians	**E**	Document analysis	No	-	-	0	Human	Regional	Yes
Veterinarian(s)	**E**	Document analysis	No	-	-	0	Animal	Local	Yes
Veterinary Institutes	**A**	Snowballing	No	-	-	0	Animal	National	No
WHO	**B**	Document analysis	No	-	-	0	Human	International	Yes
WOAH	**B**	Document analysis	No	-	-	0	Animal	International	Yes
Zoo	**B**	Snowballing	No	-	-	0	Animal	Regional	No

A) Overarching stakeholder groups. B) Stakeholder organisations. C) Stakeholder sub-organisations. D) Stakeholder departments. E) Stakeholder individuals or professions.

* One person was interviewed about their work in two organisations. ID, Infectious Diseases.

Additional file 1C - Interview guide(s)

The semi-structured stakeholder interviews were conducted in two rounds. During the first-round interviews were held by two interviewers HB and PdB, using interview guide round 1. After the first round of interviews a second round of interviews was conducted to increase the representation of the animal health domain and because of scheduling issues with some of the previously invited organisations. Interviews in the second round were conducted by one interviewer PdB. During the second round of interviews a slightly shortened version of the original interview guide was used (interview guide –interview round 2). The interview guide was adapted based on a first analysis of the data and to reduce the interview length.

Interview guide - Interview round 1.

Interview guide: Mosquito and One Health PACT

Background: This study is part of the One Health PACT a project within the NCOH and the Wageningen University.

Goal: We aim to better understand the central issues related to the organization of mosquito-borne diseases and the coordination among all involved parties in the Netherlands.

Practical: The interview will take about 1.5 hours. Halfway through we have planned a break of 5 minutes, please let us know if you want to skip the break or if you need a break at any point during the interview. The results of this study will be analysed at organisational level without mentioning any names of the interviewees. In addition to that, no quotes that give away the identity of the interviewee will be used. We would like to record this interview so that we can further supplement our notes with the recording. Do you agree with the recording? If so, I will start the recording now.

Question 1: Can you tell us about your daily work? What does your day typically involve?

- How long have your worked for XXX
- How long have you been in this position?

Question 2: How does your work relate to Mosquito-borne diseases? Or, if work isn't related to mosquito-borne diseases; how does it relate to (zoonotic) infectious diseases or healthy living environment

- What are your tasks within this function? (policy making/ facilitation/ research)

Part 2:

Question 3: What are key priorities/ goals for your organisation, related to mosquito-borne diseases?

Question 4: How do you approach open questions or information needs, regarding policy making or policy implementation, related to mosquito-borne diseases (or zoonotic infectious diseases/ healthy living environment)?

Question 5: Can you tell us a bit more about your collaborations related to infectious diseases/ mosquito-borne diseases?

	Mosquito-borne diseases		Notes
	We depend on ... for the execution of our tasks	(Others) depend on us for the execution of their tasks	
**Information (signals)**			
**Knowledge**			
**Practical execution of tasks**			
**Finances**			
**Authority**			
**Support (for decisions)**			
**Communication**			

Question 6: Can you give us an example of what the decision-making process in your organisation looks like when there is a complicated situation at hand without a clear solution?

- If problems with vectors (mosquitoes) and mosquito-borne diseases increase the coming years, do you expect this process to change? If so, how?

Question 7: According to you, what are the most significant organizational or coordination challenges for the Netherlands in the field of mosquito-borne diseases? In short, what makes it complex?

Part 3: End of the interview

We would like to thank you for your time and participation.

During the interview we filled out a table. In case you would like to go over the table again to see if you have any additions or changes, please let us know. Then we'll send the table to you.

The interview will be transcribed. The transcript will be sent to you for a final check to make sure there aren't any transcribing errors or statements we might have misinterpreted. The table will also be included in the transcript.

Interview guide - Interview round 2.

Interview study: Network analysis of actors involved in knowledge and information sharing related to mosquito-borne diseases.

Background: This study is part of the One Health PACT a project within the NCOH.
Goal: We would like to identify actors with a role in information and knowledge sharing related to mosquito-borne diseases to get an overview of the network of involved actors. Even if at this point your work is not or hardly related to this subject, we are still interested in hearing about your main tasks and collaborations.
Practical: the interview will take approximately 1 hour, please let us know if you need a break during the interview. The results of this study will be analysed at organisational level without mentioning any names of the interviewees. In addition to that, no quotes that give away the identity of the interviewee will be used.

We would like to record this interview so that we can further supplement our notes with the recording. Do you agree with the recording? If so, I will start the recording now.

Part 1: introduction

Question 1: Can you tell us about your daily work? What are your main tasks? What does your day typically involve?

- How long have your worked for XXX
- How long have you been in this position?

Question 2: How does your work relate to Mosquito-borne diseases? Or, if work isn't related to mosquito-borne diseases; how does it relate to (zoonotic) infectious diseases or healthy living environment

- What are your tasks within this function? (policy making/ facilitation/ research)
- What are your responsibilities for preparedness and response to mosquito-borne diseases?

Part 2

Question 3: Can you tell us a bit more about your (collaboration) network related to infectious mosquito-borne diseases? (or zoonotic infectious diseases/ healthy living environment)

While we are discussing this question, I would like to fill out the table.

	**Mosquito-borne diseases**		**Notes**
	We depend on ... for the execution of our tasks	(Others) depend on us for the execution of their tasks	
**Information (signals)**			
**Knowledge**			
**Practical execution of tasks**			
**Communication**			

- How do you reach out to these actors? How do these actors reach you? (regular meeting structure, direct contact, indirect contact?)
- For how long have you been working together with this organisation?
- What is the collaboration like? Is it through a regular meeting structure, direct/ indirect contact

Are there any meetings, networks, or knowledge platforms related to the subject mosquito-borne diseases, that you or your organisation are involved in?

Question 4: How do you approach open questions or information needs, regarding policy making or policy implementation (related to mosquito-borne diseases/ zoonotic infectious diseases/ healthy living environment)?

- Does the information/ knowledge that is currently available to you, give you sufficient insights to make decisions on for instance policy?
- What kind of additional information would help you get a better understanding of situation, and might help you make decisions? (this can be any kind of information, even if it is currently not available or possible to obtain this)
- How would this information (knowledge) change your actions?

Question 5: According to you, what are the most significant organizational or coordination challenges for the Netherlands in the field of mosquito-borne diseases? In short, what makes it complex?

Part 3: End of the interview

We would like to thank you for your time and participation.

During the interview we filled out a table. In case you would like to go over the table again, to see if you have any additions or changes, please let us know. Then we'll send the table to you.

The interview will be transcribed. The transcript will be sent to you for a final check to make sure there aren't any transcribing errors or statements we might have misinterpreted. The table will also be included in the transcript.

Additional file 1D– Stakeholder centrality tables

Stakeholders' positions within each network plot were assessed by calculating closeness- and betweenness centrality. Closeness centrality measures the distance of one stakeholder to all other stakeholders in the network reflecting how quickly for example, information, can spread from a certain stakeholder to all others. Closeness centrality ranges between 0 and 1 and is calculated using the following formula (Igraph R).[11] $C(v)\frac{1}{\sum u \neq v^{d(u,v)}}$

Where C(v) is the closeness centrality of stakeholder v, which is calculated using the harmonic mean of the shortest path length from a stakeholder (v) to all other stakeholder (u) in the network, excluding the stakeholder v itself. Stakeholders with a closeness centrality close to 1 can share information to all other stakeholders in the network with minimal intermediary steps.

Betweenness centrality measures how often a stakeholder is the shortest way to connect two other unconnected stakeholders in the network. The range of betweenness centrality can differ greatly depending on the size of the network, therefore scores were normalized (range 0-1) to allow for easier interpretation. Normalized betweenness centrality is calculated using the following formulas (Igraph R).[11] $B(v) = \sum s \neq v \neq t \frac{\sigma st(v)}{\sigma st}$

Where B(v) is the betweenness centrality of stakeholder v, which is calculated by summing, over all pairs of stakeholders (s and t) excluding v, the number of shortest paths from s to t that pass-through stakeholder v. This centrality score is then normalized so values range between 0-1 using this formula $N\ B(v)\frac{B(v)}{(N-1)(N-2)}$

Where N is the total number of stakeholders in the network. Stakeholders with a high betweenness centrality are the shortest path between multiple otherwise unconnected nodes and might act as intermediaries in transferring e.g. information between these less connected parts of the network. All stakeholder centrality values are interpreted in relative comparisons within the same network, by identifying the highest and lowest centrality values when compared to the other stakeholders in that specific network.

Network analysis revealed two isolated networks for knowledge sharing and one isolated network for collaborations. R and Cytoscape analysed these networks as separate networks, since these networks only contain two stakeholders this results in a closeness centrality score of one. Additionally, because of the directionality of the knowledge network, some stakeholders are unable to reach all stakeholders in the network. However, their closeness centrality score is still calculated based on the stakeholders they can

reach, which leads to an overestimation of their centrality scores. The closeness centrality scores of these stakeholders cannot be interpreted and are highlighted in gray (table 2).

Table 2. Closeness centrality (range 0-1) and normalized (N) betweenness centrality (range 0-1) scores of each stakeholder shown per domain and governance level for the three different interaction types: Information sharing; Knowledge sharing; and Collaboration.

			**Information sharing**		**Knowledge sharing**		**Collaborations**	
			Closeness Centrality	N Betweenness Centrality	Closeness Centrality	N Betweenness Centrality	Closeness Centrality	N Betweenness Centrality
**Human Domain**	**International**	ECDC	0.3425	0.0748	1.0000	0.0000	0.2821	0.0000
		International Blood Bank(s)	-	-	1.0000	0.0000	-	-
		WHO	-	-	-	-	0.2821	0.0000
	**National**	Association of public health and safety services	-	-	-	-	-	-
		Blood Bank	0.3448	0.0000	1.0000	0.0000	0.4151	0.0435
		Ministry of Health	1.0000	0.0010	1.0000	0.0062	0.3860	0.1540
		National Public Health Institute [ID]	0.4237	0.1664	0.7778	0.0531	0.5641	0.1928
		Patient Organisation(s)	0.0000	0.0000	-	-	-	-
	**Regional**	Academic Collaborative Centre	-	-	-	-	-	-

		Academic Hospitals	-	-	-	-	-	-
		Hospitals	0.0000	0.0000	-	-	-	-
		Microbiology labs	0.2874	0.0000	-	-	-	-
		Municipal (public) health service [ID]	0.3906	0.1337	0.5833	0.0023	0.4314	0.0761
		Secondary Care Physicians	0.3571	0.0252	-	-	-	-
	**Local**	Civil Society Organisations	-	-	-	-	-	-
		General Practitioner(s)	0.2841	0.0000	-	-	-	-
		Health Organisations	-	-	-	-	-	-
		Municipality [Health]	-	-	-	-	-	-
**Animal Domain**	**International**	WOAH	0.0000	0.0000	-	-	-	-
	**National**	Animal health service (GD)	0.3012	0.0801	1.0000	0.0000	0.3385	0.0169
		Bioveterinary Research (laboratory)	1.0000	0.0252	0.4000	0.0000	0.4231	0.0097
		Dutch Veterinary Association	-	-	0.0000	0.0000	-	-
		Food and Consumer Product Safety Authority	0.3425	0.0716	0.5833	0.0015	0.4400	0.0688
		Horse Sector Association	0.0000	0.0000	-	-	-	-
		Knowledge Institute (Sea Research)	-	-	-	-	-	-
		Knowledge Institute (Bird Research)	0.2453	0.0000	-	-	-	-
		Mammal Association	-	-	-	-	-	-
		Research Institute (Ecology and Birds)	0.3125	0.0433	0.4167	0.0000	0.3099	0.0000
		University (Utrecht)	-	-	-	-	0.3333	0.0169
		Veterinary Institutes	-	-	-	-	-	-
		National Wildlife Health Centre	0.3846	0.0313	-	-	0.4681	0.0353
	**Regional**	Animal Shelters	-	-	-	-	-	-
		Zoo	-	-	-	-	-	-

	**Local**	Bird Ringers	0.0000	0.0000	-	-	-	-
		Hunter Organisation	-	-	-	-	-	-
		Veterinarian(s)	0.2336	0.0000	-	-	-	-
**Vector Domain**	**National**	National Centre Vector Monitoring	0.4902	0.0842	0.6364	0.1123	0.5500	0.2281
		Knowledge Institute (Insects)	-	-	-	-	-	-
		Pest Control Company	0.2660	0.0020	0.5185	0.0508	0.3607	0.0000
		Platform Invasive Species	0.0000	0.0000	-	-	-	-
		University (Leiden)	0.2941	0.0000	-	-	0.3929	0.0000
		University (Wageningen)	0.4098	0.0953	1.0000	0.0092	0.5641	0.2239
**Environment Domain**	**International**	EEA (European Environmental Agency)	-	-	-	-	-	-
	**National**	Association of Water Authorities	-	-	-	-	-	-
		Delta Commissioner	-	-	-	-	-	-
		Environmental Assessment Agency	-	-	-	-	-	-
		Infrastructure and Water Mangement	-	-	-	-	-	-
		Knowledge Institute (water and subsurface)	-	-	0.4054	0.0000	-	-
		Knowledge Institute (Water Authorities)	-	-	-	-	-	-
		Ministry Economics and Climate	-	-	-	-	-	-
		National Public Health Institute [Environment]	-	-	-	-	-	-
		Nature Organisation(s)	-	-	-	-	-	-
		Research Institute (Living Environment)	-	-	-	-	-	-
		Research Institute (National Meteorological)	-	-	0.4054	0.0000	-	-
		Research institute (Human demographics)	-	-	-	-	-	-
		Research Institute (Biology and Environment)	-	-	-	-	-	-
	**Regional**	Dutch Water Authorities	-	-	0.0000	0.0000	-	-

		Environment Agencies	-	-	-	-	-	-
		Port (Rotterdam)	-	-	-	-	-	-
		Province(s)	-	-	-	-	-	-
		Municipal (public) health service [Environment]	0.0000	0.0000	0.5833	0.0062	0.3056	0.0000
	**Local**	Agriculture sector	-	-	-	-	-	-
		Environmental Developers	-	-	-	-	-	-
		Housing Corporation(s)	-	-	0.0000	0.0000	-	-
		Land Management Organisations	-	-	-	-	-	-
		Municipality [Environment]	0.0000	0.0000	0.0000	0.0000	1.0000	0.0000
		Parks Department	-	-	-	-	-	-
		Project Developers	-	-	-	-	-	-
**Human-Animal**	**International**	Reference laboratories	0.6000	0.0000	-	-	-	-
	**National**	Erasmus Medical Centre (Arbovirus reference laboratory)	0.4310	0.1488	-	-	0.4400	0.0779
		University (Rotterdam)	0.2941	0.0000	-	-	0.4314	0.0833
**Other Domain**	**International**	EASIN (EU. Alien Species)	-	-	-	-	-	-
		European Union	-	-	-	-	-	-
		International Governments	-	-	-	-	0.3607	0.0000
		International Partners	0.6667	0.0000	0.5714	0.0062	0.5116	0.1721
	**National**	Academia	-	-	0.4054	0.0000	0.4583	0.0174
		Central Government	-	-	0.0000	0.0000	-	-
		Consulting Companies	-	-	-	-	-	-

		Database (Flora and Fauna)	-	-	-	-	-	-
		Database (National Statistics)	-	-	-	-	-	-
		Knowledge institute(s)	-	-	-	-	-	-
		Ministry Agriculture and Nature	0.0000	0.0000	0.0000	0.0000	-	-
	**Regional**	Safety and Crisis Management	-	-	-	-	-	-
	**Local**	Citizens	0.3676	0.0665	0.0000	0.0000	-	-
		Local Enterprise(s)	-	-	0.0000	0.0000	-	-
		Resident Association(s)	-	-	-	-	1.0000	0.0000
	**Other**	Private Companies	-	-	0.3500	0.0000	0.3667	0.0000

[ID] = Infectious diseases. Grey values are an overestimation due to network characteristics and should not be interpreted.

Additional file 1E – Detailed stakeholder network figures

Figure 2. "Mosquito and Mosquito-borne virus" interaction networks between the domains.

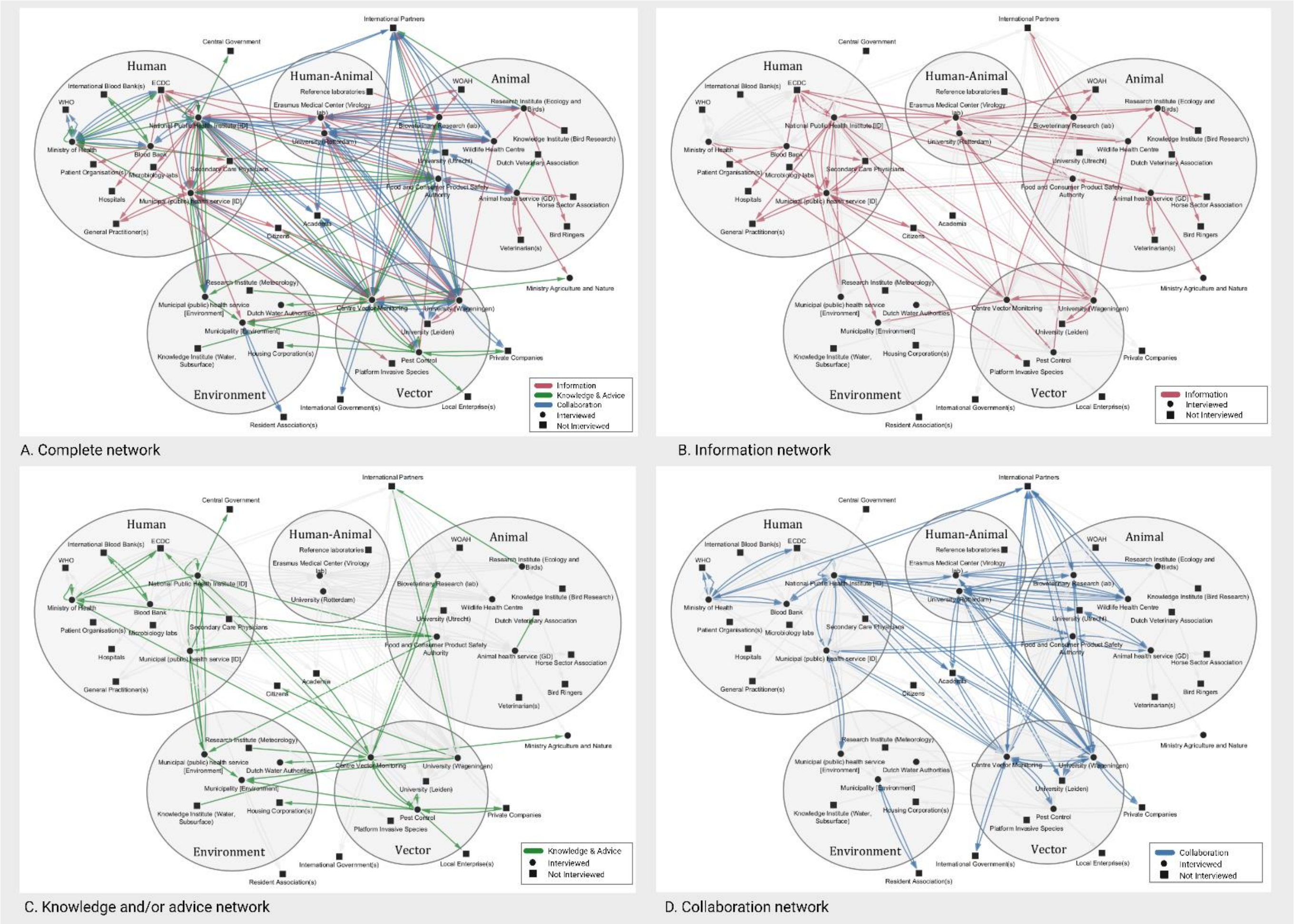

Panel A shows the complete network for all connections between the different domains (human, animal, human-animal, vector, environment and ‘other’). Panel B. Shows information sharing (in pink). Panel C. shows knowledge and or advice sharing (in green). Panel D. shows collaborations (in blue). Stakeholders outside of the domain circles are classified as other.

Figure 3. “Mosquito and Mosquito-borne virus” and “scale-up” interaction networks between the domains.

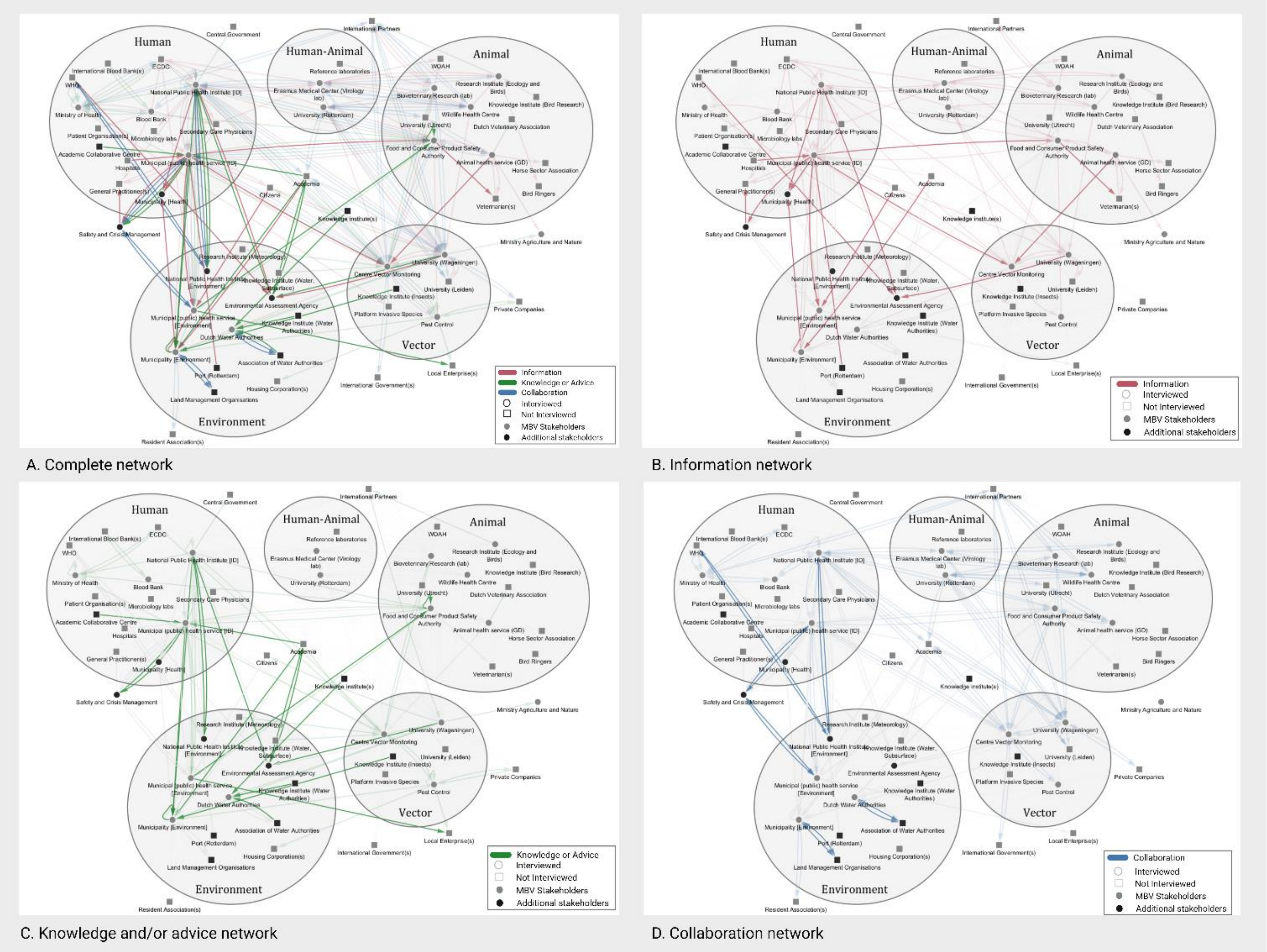

Panel A shows the complete network for all connections between the different domains (human, animal, human-animal, vector, environment and 'other'). Panel B. Shows information sharing (in pink). Panel C. shows knowledge and or advice sharing (in green). Panel D. shows collaborations (in blue). Stakeholders outside of the domain circles are classified as other. Darker lines are connections in case of "scale-up", lighter lines are MBV connections.

Figure 4. “Mosquito and Mosquito-borne virus” and “(zoonotic) infectious diseases” interaction networks between the domains.

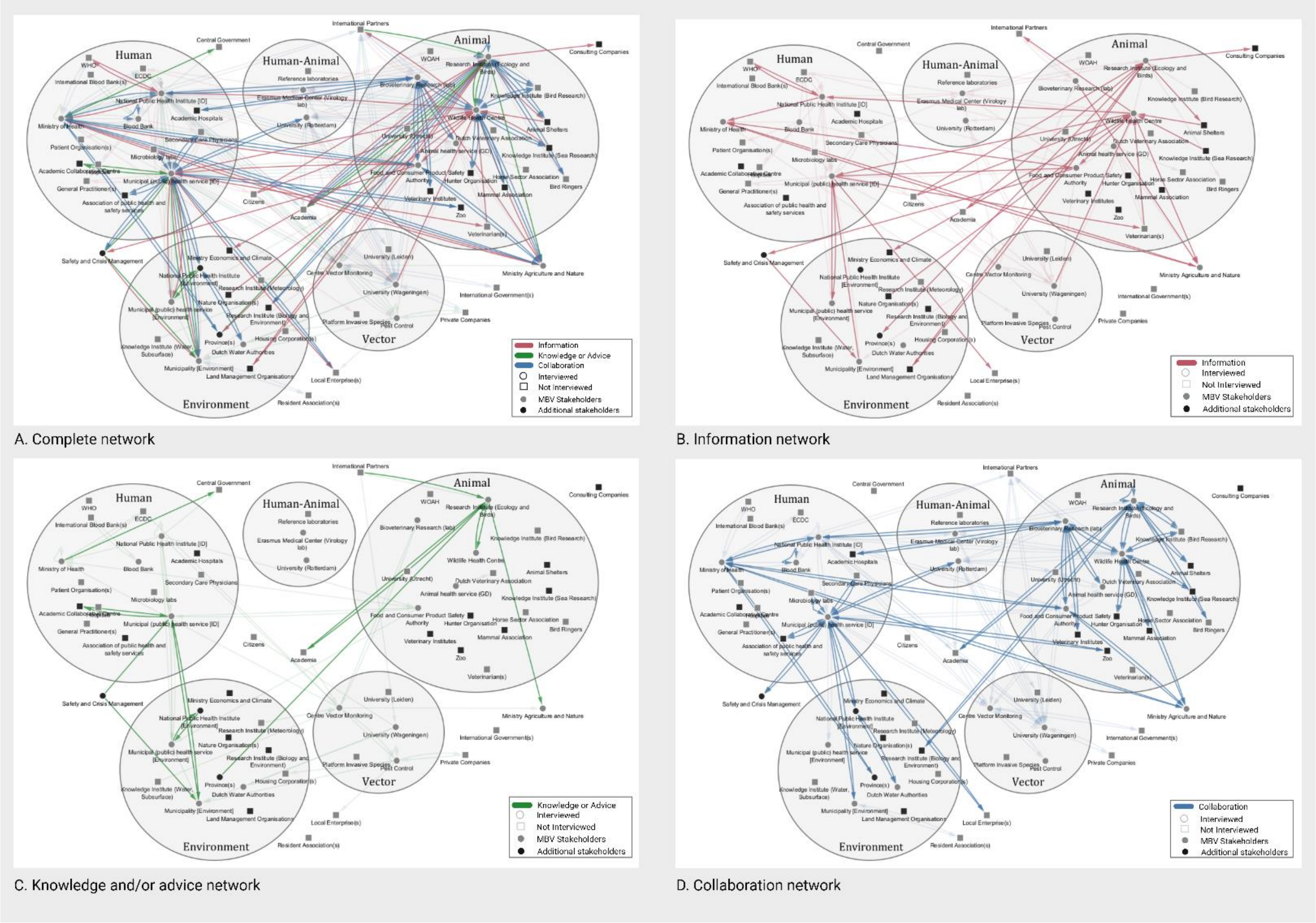

Panel A shows the complete network for all connections between the different domains (human, animal, human-animal, vector, environment and ‘other’). Panel B. Shows information sharing (in pink). Panel C. shows knowledge and or advice sharing (in green). Panel D. shows collaborations (in blue). Stakeholders outside of the domain circles are classified as other. Darker lines are “zoonotic infectious diseases” connections, lighter lines are MBV connections.

Figure 5. “Healthy living environment and climate adaptation” interaction networks between the domains.

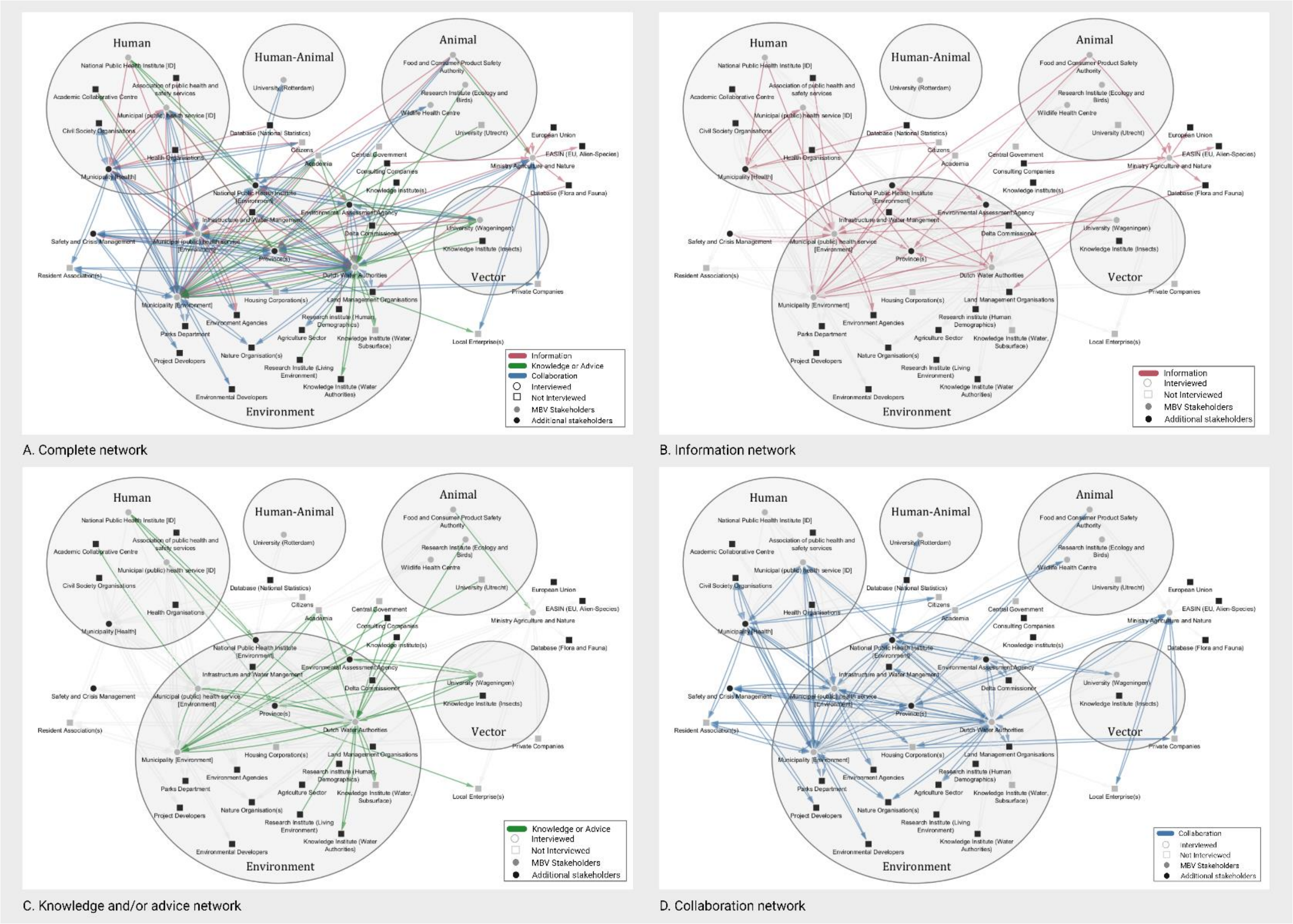

Panel A shows the complete network for all connections between the different domains (human, animal, human-animal, vector, environment and 'other'). Panel B. Shows information sharing (in pink). Panel C. shows knowledge and or advice sharing (in green). Panel D. shows collaborations (in blue). Stakeholders outside of the domain circles are classified as other.

References:

1. Yvonne Huizer KH AJ, Corien Swaan, Ronald Reekers, Rob van Kessel, Ronald ter Schegget, Reinoud Wolter: **General protocol infectious diseases [Generaal draaiboek infectieziekten]**. In. Website National Institute for Public Health and the Environment [RIVM]; 2014.
2. (RIVM) NIfPHatE: **International Health Regulations [Internationale Gezondheidsregeling]**. In. Website National Institute for Public Health and the Environment (RIVM); 2009.
3. Peter Jacobs RvK, Mauro de Rosa, Ife Slegers - Fitz-James, Charlotte Verbart: **Vademecum Zoonoses [Vademecum zoönosen]**. In. National Institute for Public Health and Environment [Rijksinstituut voor Volksgezondheid en Milieu (RIVM)]; 2021.
4. Sabine Bantjes MB: **Exotic Mosquitoes - Policy for invasive mosquitoes in the Netherlands [Exotische steekmuggen - Beleid bij invasieve exotische steekmuggen in Nederland]**. In. Webpage National Institute for Public Health and the Environment (RIVM); 2021.
5. Koopmans MPG: **Preparing for vector-borne virus outbreaks in a changing world: a One Health Approach** In.: Dutch National Research Agenda, Research along Routes by Consortia, Full proposal form 2018, Version 20181010. (Not published online). Netherlands Organisation for Scientific Research; 2018.
6. **Public Health Act [Wet publieke gezondheid]**. In: *BWBR0024705.* Netherlands; 2021.
7. **International Health Regulations [nternationale Gezondheidsregeling (2005), Genève, 23-05-2005]**. In.; 2005.
8. **Municipality Act [Gemeentewet]** In: *BWBR00054116.* Netherlands; 2021.
9. **Animal Health and Welfare Act [Gezondheids- en welzijnswet voor dieren]**. In: *BWBR0005662.* 2019.
10. **Environment and Planning Act [Omgevingswet]**. In. Netherlands; In development.

11. Csárdi G NT, Müller K, Horvát S, Traag V, Zanini F, et al.: **graph for R: R interface of the igraph library for graph theory and network analysis**. In. Zenodo; 2023.
